# Supplementary material for: Economic evaluation of the OSAC randomised controlled trial: oral corticosteroids for non-asthmatic adults with acute lower respiratory tract infection in primary care
Source: BMJ Open. 2020 Feb 18;10(2):e033567. doi: 10.1136/bmjopen-2019-033567 (PMC7045138; doi:10.1136/bmjopen-2019-033567)
Supplement: Supplementary data [file bmjopen-2019-033567supp001.pdf]

Appendix

Table A. Participants' baseline characteristics

	Prednisolone (N=198)	Placebo (N=200)
Centre, n (%)		
Bristol	118 (60%)	113 (57%)
Oxford	39 (20%)	45 (23%)
Southampton	24 (12%)	21 (11%)
Nottingham	17 (9%)	21 (11%)
Demographics and past medical history		
Gender, n (%) male	82 (41%)	66 (33%)
Age, mean (SD)	50.0 (16.1)	44.8 (15.5)
Weight kg, median (IQR)[a]	77.0 (64.5,91.0)	76.0 (66.5,90.5)
Height cm, median (IQR)[b]	168.0 (161.0,175.0)	168.0 (163.0,176.0)
Ethnicity, n (%) white[c]	188 (95%)	193 (97%)
Occupation, n (%)		
Employed	137 (69%)	143 (72%)
Unemployed	17 (9%)	21 (11%)
Retired	41 (21%)	30 (15%)
Full-time education	3 (2%)	6 (3%)
Deprivation (IMD)[d], median (IQR)[e]	11.0 (5.0,23.0)	12.0 (5.0,23.0)
Smoking status, n (%)[f]		
Current	31 (16%)	38 (19%)
Past	63 (32%)	55 (28%)
Never	104 (53%)	106 (53%)
Lives with smoker, n (%)[g]	25 (14%)	32 (16%)
Received asthma medication >5 years previously[h]	10 (5%)	8 (4%)
Personal history of hay fever[i]	41 (22%)	46 (24%)
Personal history of eczema[j]	30 (16%)	26 (14%)
Family history asthma/hay fever/eczema, n (%)[k]	73 (40%)	76 (40%)
Influenza vaccine in last 12 months, n (%)	63 (32%)	44 (22%)
Recruited in winter (1st Oct-31st March)	112 (57%)	114 (57%)
Clinical characteristics and management		
Prior duration of cough, median (IQR) days	13.0 (7.0,20.0)	10.0 (6.0,17.5)
Sputum (symptom <24hr), n (%)[l]	149 (76%)	156 (78%)
Shortness of breath (symptom <24hr) n (%)	146 (74%)	133 (67%)

(Table continues on next page)

Moure-Fernandez A, *et al. BMJ Open* 2020; 10:e033567. doi: 10.1136/bmjopen-2019-033567

Table A. Participants' baseline characteristics *(cont.)*

	**Prednisolone** (N=198)	**Placebo** (N=200)
Wheeze (symptom <24hr), n (%)[l]	88 (45%)	98 (49%)
Chest pain (symptom <24hr) n (%)	88 (44%)	97 (49%)
Patient reported illness severity (0-10), median (IQR)[m]	6.0 (5.0,7.0)	5.0 (4.0,7.0)
Pulse rate (bpm), mean (SD)	77.8 (12.3)	77.7 (11.8)
Temperature (^{o}C), mean (SD)	36.6 (0.5)	36.6 (0.4)
Oxygen saturation (%), mean (SD)[n]	97.5 (1.3)	97.8 (1.1)
Baseline abnormal peak flow[o]	87 (44%)	79 (40%)
Abnormal respiratory rate, n (%)	2 (1%)	1 (1%)
Chest recession/prolonged expiration	0 (0%)	1 (1%)
Wheeze/rhonchi (auscultation), n (%)	11 (6%)	11 (6%)
Crackles/crepitations (auscultation), n (%)[p]	4 (2%)	6 (3%)
Bronchial breathing	0 (0%)	2 (1%)
Taken prescribed β agonist in past 24 hours, n (%)	9 (5%)	3 (2%)
OTC[q] drugs taken for current cough, n (%)	128 (65%)	139 (70%)
Given delayed antibiotic script, n (%)	22 (11%)	25 (13%)

[a] Weight missing for 2 prednisolone participants
[b] Height missing for 1 prednisolone participant
[c] Ethnicity missing for 1 placebo participant
[d] English Index of Multiple Deprivation scores (2015) [Geoconvert: UK Data Service Census Support]
[e] IMD missing for 2 prednisolone and 7 placebo participants
[f] Smoking status missing for 1 placebo participant
[g] Living with smoker missing for 15 prednisolone and 5 placebo participants
[h] Personal history of asthma missing for 10 prednisolone patients and 7 placebo patients
[I] Personal history of hayfever missing for 10 prednisolone patients and 11 placebo patients
[j] Personal history of eczema missing for 14 prednisolone patients and 10 placebo patients
[k] Family history of hay fever/eczema/asthma missing for 16 prednisolone and 11 placebo participants
[l] Sputum and wheeze presence in 24 hours, missing for 1 prednisolone participant
[m] Patient reported illness severity measured on zero to 10 scale, missing for 1 prednisolone participant
[n] Oxygen saturation missing for 1 prednisolone participant
[o] Baseline abnormal peak flow (defined as <80% of expected peak flow) was missing for 1 prednisolone patient
[p] Includes unilateral and bilateral
[q] Over-the-counter

Table B. Percentage of patients reporting no problems in response to the five domains of the EQ-5D

		**Baseline – all participants** (n=199 placebo; 198 prednisolone)	**Baseline – participants with week 4 data** (n=171 placebo; 177 prednisolone)	**Week 4** (n=171 placebo; 177 prednisolone)
Mobility	Placebo	93.0	94.1	94.7
	Prednisolone	91.9	91.5	94.4
Self-care	Placebo	97.5	97.7	97.1
	Prednisolone	99.0	98.9	99.4
Usual activities	Placebo	89.9	90.0	83.0
	Prednisolone	82.3	82.5	93.2
Pain/discomfort	Placebo	61.3	62.9	81.3
	Prednisolone	55.6	54.8	87.6
Anxiety/depression	Placebo	83.4	84.1	86.5
	Prednisolone	82.3	82.5	89.3

Moure-Fernandez A, *et al. BMJ Open* 2020; 10:e033567. doi: 10.1136/bmjopen-2019-033567
