# Supplementary material for: Economic evaluation of the OSAC randomised controlled trial: oral corticosteroids for non-asthmatic adults with acute lower respiratory tract infection in primary care
Source: BMJ Open. 2020 Feb 18;10(2):e033567. doi: 10.1136/bmjopen-2019-033567 (PMC7045138; doi:10.1136/bmjopen-2019-033567)
Supplement: Author's manuscript [file bmjopen-2019-033567.draft_revisions.pdf]

BMJ Open

BMJ Open is committed to open peer review. As part of this commitment we make the peer review history of every article we publish publicly available.

When an article is published we post the peer reviewers' comments and the authors' responses online. We also post the versions of the paper that were used during peer review. These are the versions that the peer review comments apply to.

The versions of the paper that follow are the versions that were submitted during the peer review process. They are not the versions of record or the final published versions. They should not be cited or distributed as the published version of this manuscript.

BMJ Open is an open access journal and the full, final, typeset and author-corrected version of record of the manuscript is available on our site with no access controls, subscription charges or pay-per-view fees (http://bmjopen.bmj.com).

If you have any questions on BMJ Open's open peer review process please email info.bmjopen@bmj.com

BMJ Open

Cost-effectiveness analysis of oral corticosteroids for non-asthmatic adults with acute lower respiratory tract infection in primary care: economic evaluation of the OSAC randomised controlled trial

Journal:	*BMJ Open*
Manuscript ID	bmjopen-2019-033567
Article Type:	Original research
Date Submitted by the Author:	11-Aug-2019

Complete List of Authors:	Moure-Fernandez, Aide; Hospital Clínic de Barcelona,<br>Hollinghurst, Sandra; University of Bristol, Community Based Medicine<br>Carroll, Fran; University of Bristol, School of Social and Community Medicine<br>Downing, Harriet; University of Bristol, Community Based Medicine<br>Young, Grace; University of Bristol, School of Social and Community Medicine; Bristol Randomised Trials Collaboration, University of Bristol<br>Brookes, Sara ; University of Bristol,<br>May, Margaret; University of Bristol, School of Social and Community Medicine<br>El-Gohary, Magdy; University of Southampton, 2) Department of Primary Care and Population Sciences<br>Harnden, Anthony; Oxford University, Dept of Primary Health Care<br>Kendrick, Denise; University of Nottingham<br>Lafond, Natasher; University of Nottingham School of Medicine<br>Little, Paul; University of Southampton, Primary Care and Population Science<br>Moore, Michael; University of Southampton Medical School, Primary Care Medical Group<br>Orton, Elizabeth; University of Nottingham<br>Thompson, Matthew; University of Washington, Department of Family Medicine<br>Timmins, David; Oxford Brookes University Faculty of Health and Life Sciences, NIHR School for Primary Care Research; Nuffield department of primary care health sciences<br>Wang, Kay; Nuffield Department of Primary Care Health Sciences<br>Hay, Alastair; University of Bristol, Centre for Academic Primary Care

Keywords:	HEALTH ECONOMICS, RESPIRATORY MEDICINE (see Thoracic Medicine), cost-effectiveness, ALRTI, PRIMARY CARE

SCHOLARONE™
Manuscripts

Cost-effectiveness analysis of oral corticosteroids for non-asthmatic adults with acute lower respiratory tract infection in primary care: economic evaluation of the OSAC randomised controlled trial

Aída Moure Fernández; Sandra Hollinghurst; Fran Carroll; Harriet Downing; Grace Young; Sara Brookes; Margaret May; Magdy El-Gohary; Anthony Harnden; Denise Kendrick; Natasher Lafond; Paul Little; Michael Moore; Elizabeth Orton; Matthew Thompson; David Timmins; Kay Wang; Alastair D Hay.

Author Affiliations: Centre for Academic Primary Care, National Institute for Health Research (NIHR) School for Primary Care Research, Population Health Sciences, Bristol Medical School, University of Bristol, Bristol, England (Hay, Hollinghurst, Downing); Primary Care and Population Science, NIHR School for Primary Care Research, Faculty of Medicine, University of Southampton, Aldermoor Health Center, Southampton, England (Little, El-Gohary, Moore); Nuffield Department of Primary Care Health Sciences, NIHR School for Primary Care Research, University of Oxford, Oxford, England (Harnden, Wang, Timmins); Department of Family Medicine, University of Washington, Seattle (Thompson); Division of Primary Care, NIHR School for Primary Care Research, School of Medicine, University of Nottingham, Nottingham, England (Kendrick, Orton, Lafond); Population Health Sciences, Bristol Medical School, University of Bristol, Bristol, England (Brookes, May); Bristol Randomised Trials Collaboration, Population Health Sciences, Bristol Medical School, University of Bristol, Bristol, England (Young); Oblikue Consulting, SL (Moure Fernández); Translational Health Sciences, Bristol Medical School, University of Bristol, Bristol, England (Carroll).

For peer review only - http://bmjopen.bmj.com/site/about/guidelines.xhtml
For peer review only

Corresponding Author: Aída Moure Fernández. Hospital Clínic de Barcelona. Villarroel, 170. 08036 Barcelona, Spain.

For peer review only

For peer review only - http://bmjopen.bmj.com/site/about/guidelines.xhtml

Abstract

Objective

To estimate the costs and outcomes associated with treating non-asthmatic (nor suffering from other lung-disease) adults presenting to primary care with acute lower respiratory tract infection (ALRTI) with oral corticosteroids compared with placebo.

Design

Cost-consequence analysis alongside a randomised controlled trial. Perspectives included the healthcare provider, patients, and productivity losses associated with time off work.

Setting

Fifty-four NHS general practices in England.

Participants

398 adults attending NHS primary practices with ALRTI but no asthma or other chronic lung disease, followed up for 28 days.

Interventions

2x20mg oral prednisolone daily for five days versus matching placebo tablets.

Outcome measures

Quality-adjusted life years using the EQ-5D-5L measured weekly; duration and severity of symptom. Direct and indirect resources related to the disease and its treatment were also collected. Outcomes were measured for the 28-day follow up.

Results

198 (50%) patients received the intervention (prednisolone) and 200 (50%) received placebo. NHS costs were dominated by primary care contacts, higher with placebo than with prednisolone (£13.11 vs £10.38) but without evidence of a difference (95% CI: -£3.05 to £8.52). The trial medication cost of £1.96 per patient would have been recouped in

For peer review only - http://bmjopen.bmj.com/site/about/guidelines.xhtml

prescription charges of £4.30 per patient overall (55% participants would have paid £7.85), giving an overall mean ‘profit’ to the NHS of £7.00 (95% CI: £0.50 to £17.08) per patient. There was a QALY gain of 0.03 (95% CI: 0.01 to 0.05) equating to half a day of perfect health favouring the prednisolone patients; there was no difference in duration of cough or severity of symptoms.

Conclusions

The use of prednisolone for non-asthmatic adults with ALRTI, provided small gains in quality of life and cost savings driven by prescription charges. Considering the clinical and cost-effectiveness results and possible side effects of corticosteroids, the short-term benefits may not outweigh the long term harms.

For peer review only

Strengths and limitations of this study

- The economic evaluation was part of a rigorously conducted multicentre randomised controlled trial, involving a representative population of patients not thought to need immediate antibiotic treatment.
- The economic evaluation included the perspectives of patients and time off work as well as the NHS.
- Low levels of missing cost and outcome data, with EQ-5D observations from multiple time points, achieving an accurate profile of patient health-related quality of life over the period of the illness.
- The analysis was thorough and included multiple imputation of missing data and extensive sensitivity analysis.

For peer review only - http://bmjopen.bmj.com/site/about/guidelines.xhtml

Cost-effectiveness analysis of oral corticosteroids for non-asthmatic adults with acute lower respiratory tract infection: economic evaluation of the OSAC randomised controlled trial

INTRODUCTION

Acute lower respiratory tract infection (ALRTI), with symptoms such as wheeze, phlegm, and chest pain, is one of the most common reasons for patients to consult in primary care.[1] In the UK ALRTI costs the National Health Service at least £190 million annually,[2] and further costs are borne by patients in self-managing their condition[3, 4] and by society in general because of work absenteeism.[5]

Despite lack of evidence of efficacy and the recommendation from the National Institute for Health and Care Excellence of a non-antibiotic pathway,[6] antibiotics are still frequently prescribed to treat ALRTI.[7, 8] This unnecessary prescribing fuels antimicrobial resistance, raises patient expectations for similar treatment in the future (so-called 'illness medicalisation'), and is a wasteful use of healthcare resources.

Oral and inhaled corticosteroids are widely used to treat symptoms of asthma[9] and there is some evidence of the effectiveness of high doses of inhaled corticosteroids in reducing cough frequency among non-smokers with ALRTI who do not have asthma or other chronic lung disease.[10] Given the similarity in symptoms of ALRTI and those of asthma, we tested the hypothesis that corticosteroids might be an effective treatment for ALRTI in adults without asthma or other chronic lung disease. Indeed, there is increasing evidence from Europe[11] and the United States[12] of steroid prescribing for patients with ALRTI, with a

For peer review only - http://bmjopen.bmj.com/site/about/guidelines.xhtml

secondary analysis of one US study[12] showing 15% of non-asthmatic adults with ALRTI being prescribed oral steroids.

The OSAC study[13,14] examined the effectiveness and cost-effectiveness of oral corticosteroids (more specifically oral prednisolone) in treating ALRTI in a non-asthmatic adult population compared with placebo. Here, we report on the results of the economic evaluation.

METHODS

The OSAC study was a two-arm randomised controlled trial that aimed to test the provision of oral corticosteroids to adults with ALRTI but no asthma or other chronic lung disease such as chronic obstructive pulmonary disease. Details of the trial procedures and clinical results are reported elsewhere.[13,14] Briefly, patients with an ALRTI-related cough were recruited from 54 primary care practices across four areas of England and randomly allocated to receive either oral prednisolone (intervention) or placebo (control) for their acute cough symptoms. The intervention consisted of 40mg (two 20mg tablets) of prednisolone daily for five days. The control group received matching placebo tablets. The primary clinical outcomes of the study were duration of “moderately bad or worse” cough, and symptom severity for days 2 to 4 post randomisation. The primary outcome for the economic evaluation was quality adjusted life years (QALYs) which were estimated from responses to the EQ-5D-5L.[15]

For peer review only - http://bmjopen.bmj.com/site/about/guidelines.xhtml

Design

The economic evaluation was conducted from the perspectives of the NHS, patients, and a broader perspective taking into account differences in work-related productivity costs; we included all resource use during the 28-day follow up period. We used a cost-consequences design,[16] comparing cost from the three perspectives with the clinical outcomes and QALYs, in order to see all possible costs and health consequences.

Identification of resources

NHS resources were identified as being: the trial medication, primary care consultations, other relevant prescribed medication, hospital care, use of NHS 111, and ALRTI-related investigations such as chest X-ray. Patient resource use was identified as being: travel, prescription costs, and over-the-counter medication and remedies. Productivity losses were identified as being days off work due to the cough.

Measurement of resources: data collection

A primary care notes review took place at the end of the study period and data were extracted on: all primary care consultations, categorised as doctor or nurse, face to face, telephone or home visit, and in-hours or out-of-hours; prescribed medication; and investigations. Participants in the study were issued with a daily diary to complete, which was used to collect data on health service use not always reliably available from primary care notes such as use of NHS 111 and hospital services. Participant out-of-pocket expenses on travel and over-the-counter medicines were also recorded here, along with time off work. Participants were telephoned weekly to reinforce their record keeping.

For peer review only - http://bmjopen.bmj.com/site/about/guidelines.xhtml

Valuation of resources

Resources were valued as shown in Table 1. All resources were costed in pounds sterling at 2013-2014 prices, using an appropriate inflation index[17] when necessary. Costs for primary care services were obtained from Curtis;[17] calls to the NHS 111 service were costed using a published national evaluation[18] and NHS reference costs[19] were used to value secondary care services. Travelling by car was valued using the AA running schedule to cost the mileage,[20] and standard ticket prices were employed to cost the use of public transport. Productivity costs were derived using the age/sex average rate from the Annual Survey of Hours and Earnings.[21]

For prescribed medication, costs were extracted mainly from the British National Formulary (BNF).[22] A cost was obtained for each prescription based on the name of the drug, the method of administration (for example, tablet, capsule, liquid) and the number of units included in the prescription. When the cost of a drug was not available in the BNF, the Prescription Costs Analysis of England database[23] was used to cost that medication. Uncertainties around relevance (to ALRTI) and missing information were resolved by seeking clinical advice.

Careful consideration was given to the most accurate way of costing the intervention medication (prednisolone) with the aim of reflecting the true cost to the NHS if it were to be adopted as a strategy for treating ALRTI. First, thought was given to the number of pills that would be needed to cover the intervention total dose as the 20mg tablets provided in the study are not routinely available. We assumed that 5mg tablets would be a reasonable substitute, resulting in 8 tablets required per day, and 40 tablets for the duration of the

For peer review only - http://bmjopen.bmj.com/site/about/guidelines.xhtml

intervention (5 days). From the basic price of £1.03 given in the BNF, adjustments were made as follows. We subtracted the usual discount (7.61% of the basic price) eligible to the dispensing pharmacy from the manufacturer; we added a standard 90p dispensing fee and a payment of 1.24p for consumables; and as the amount required was 40 and packs contain 28 tablets, an extra 10p for splitting packs was added. This resulted in a cost for the intervention prednisolone of £1.96. To reflect a 'roll-out' situation, we allowed for potential receipts from prescription payments made by patients by including an amount for those participants who reported that they usually pay a prescription charge.

No cost was included for the placebo medication (i.e. tablets) as this was a research cost.

Measurement and valuation of quality adjusted life years

The EQ-5D-5L was completed weekly by participants from baseline during the 28-day follow up, giving us five observations to use to form QALYs. Utilities were obtained from existing preferences elicited from the general public, using the crosswalk algorithm.[15] QALYs were calculated from these utilities using the area under the curve and adjusting for baseline differences.[24]

Data analysis

Frequencies of resource use were calculated to provide a descriptive analysis of the types of resources used by primary care patients with a cough. We estimated mean resource use and cost per patient for all categories, by trial arm, to compare the prednisolone group with those using the placebo. Ordinary least squares regressions were employed to calculate the differences in costs adjusted for centre, age, gender and outcome-related baseline variables, in order to account for potential imbalances between the groups. Standard

For peer review only - http://bmjopen.bmj.com/site/about/guidelines.xhtml

deviations (for means) and bias-corrected bootstrapped confidence intervals[25] (2,000 replicates) were constructed to account for the uncertainty in the point estimates.

No discount was applied to the data as the time horizon of the study was 28 days. All analyses were carried out using Stata 13 and above.[26]

Sensitivity Analyses

Five different sensitivity analyses were conducted to test the robustness of assumptions made in the base case analysis. The effect of missing data was appraised in two scenarios using multiple imputation techniques: in addition to the imputation with chained equations (ICE) (scenario 1), which is usually employed in economic evaluations, an analysis was also carried out using the 'twofold' command in Stata; this command imputes missing values at a given time point, conditional on information at the same time point and immediately adjacent time points [27] (scenario 2), to be consistent with the methodology used in the clinical effectiveness analysis.[14] Scenario 3 excluded outpatient and A&E attendances where there was any ambiguity about their relevance to LRTI; scenario 4 used the visual analogue scale to calculate QALYs; and scenario 5 removed the prescription payments from the analysis on the basis that these are to some extent artefactual and relate specifically to England at the time of the study.

RESULTS

In total, 398 patients were included in the intention-to-treat analysis. One hundred and ninety eight (49.7%) were in the trial medication (intervention) group and 200 (50.3%) received the matched placebo (control). Baseline characteristics of the participants are

For peer review only - http://bmjopen.bmj.com/site/about/guidelines.xhtml

For peer review only

shown in Table A of the appendix. Participants were predominantly white, employed, and middle-aged. Approximately 50% in both arms stated they had never smoked. Data on NHS costs were complete for 332 (83%) participants and out-of-pocket costs for patients were reported by 329 (83%). Time off work was recorded by 321 (81%) of the participants filling the questionnaire and 346 (87%) completed the EQ-5D-5L at all five time points.

Resource use

Use of all health services was relatively modest during the 28 day follow up period. Overall just 20% participants accessed primary care, a proportion that was similar for both groups though patients in the placebo group had slightly more consultations per patient than those in the prednisolone group, which is reflected in the mean number of encounters (0.30 vs 0.27) as shown in Table 2. Prescribed medication was also slightly higher in the placebo group in terms of number of patients (15% vs 12%) being prescribed anything and the mean number of prescriptions per participant (0.22 vs 0.15). Nine participants (2%) reported using hospital services: three (1.5%) in the prednisolone group and six (3%) in the placebo group.

More placebo group participants reported buying over-the-counter medications than those in the prednisolone group (45% vs 39%) and the mean number of items per participant was higher (0.59 vs 0.48). Popular items included cough linctus and cold & flu remedies. Similarly, more placebo group participants reported time off work than prednisolone participants (32% vs 28%) and the mean length of time off was longer (1.38 days vs 0.95 days).

For peer review only

Although there was a consistently higher use of resources in the placebo group compared with the prednisolone group there is imprecision around the point estimates and, with the exception of the trial medication and associated prescription payments, there is no evidence of a difference in use between the groups as indicated by the confidence intervals reported in Table 2.

Cost analysis

Table 3 summarises the cost comparison between the two groups. The majority of NHS costs were attributable to primary care consultations with hospital visits, including for X-rays, contributing a modest amount. Over half (55%) of prednisolone participants reported that they normally pay a prescription charge; the value of prescription payments made by these patients more than covered the cost of the trial medication meaning that the NHS on balance would have made a profit. Out-of-pocket patient costs were dominated by prescription payments and the value of time off work was higher than any other category of cost. Comparing the prednisolone and placebo groups, costs were higher in the placebo group for all categories (except those related to the trial medication) though again there was no evidence of a difference between the groups as indicated by the confidence intervals.

Cost-consequence analysis

Table 4 shows the comparison of incremental costs and outcomes. Here we present the difference in cost by perspective, adjusted by centre, age, gender, and baseline covariates. The 95% confidence intervals are bootstrapped and bias corrected. The negative incremental cost of -£7.00 (95% CI: -£17.08 to -£0.50) per patient to the NHS indicates the

For peer review only - http://bmjopen.bmj.com/site/about/guidelines.xhtml

For peer review only

intervention is cheaper than placebo; it reflects the lower use of primary care by patients in the prednisolone group and the offset of intervention costs by prescription payments. From the patient perspective, despite higher travel and over the counter costs in the placebo group, total costs were approximately £3 more in the prednisolone group due to the cost of prescriptions. The value of time off work was approximately £30 higher per patient for the placebo group.

The gain in quality of life provided by the prednisolone was 0.03 QALYs (95% CI: 0.01 to 0.05) per patient, equating to about half a day of extra 'best imaginable' health during the 28 days. The percentage of patients reporting no problems for each domain of the EQ-5D are shown in table B of the Appendix. On average, the prednisolone patients improved by more than the placebo patients in all domains but the 'pain and discomfort' and 'usual activities' domains were where the greatest difference was seen.

The median duration of moderately bad or worse cough was 5 days in both groups and the hazard ratio of 1.11 (95% CI: 0.89 to 1.39) also indicates no difference. There was a reduction of 0.02 in mean symptom score for days 2 to 4 in prednisolone patients though this was less than the pre-determined clinically meaningful difference.

Sensitivity analysis

Results of the sensitivity analyses are presented in Table 5. Imputing missing data, and excluding unrelated costs make no difference to the conclusions of the base case analysis. Using the visual analogue scale (VAS) to value the QALYs reduced the QALY gain of prednisolone patients over placebo to a minimal amount (0.01: 95% CI: -0.01 to 0.03). When

For peer review only - http://bmjopen.bmj.com/site/about/guidelines.xhtml

For peer review only

prescription payments are removed the incremental cost to the NHS is still negative (-£3.04; 95% CI: -£11.31 to £5.24) though the confidence interval suggests no evidence of a true difference. The reverse is true for patient costs as placebo patients spent considerable more on travel and over-the-counter medications and remedies than those in the prednisolone group.

DISCUSSION

Summary of main findings

Prednisolone was found to be clinically ineffective for treating ALRTI in non-asthmatic adults in primary care in terms of duration of cough and symptom severity [14]; however there was evidence that patients using prednisolone experienced a greater improvement in health-related quality of life than those using the placebo. This was largely due to a greater improvement in pain/discomfort and a speedier return to carrying out usual activities. Prednisolone is a relatively inexpensive medication and, in this population of generally healthy adults ineligible for prescription charge exemption, prescription payments more than offset the cost to the NHS. Other NHS resource use was consistently higher in the placebo group across all categories though the differences were small. The value of time off work was considerable and this was higher in the placebo patients, though the limited sample size combined with patient-level variation prevented a robust conclusion.

Strengths and weaknesses

The sample included in OSAC was typical of patients consulting in primary care with ALRTI, not thought to require immediate antibiotic treatment. The two groups were well matched at baseline and there was minimal drop-out. The economic evaluation was carried out at

For peer review only - http://bmjopen.bmj.com/site/about/guidelines.xhtml

individual patient level and covered the whole 28 day period. Data on actual utility values for each group was not reported, which would have helped to understand the differences regarding quality of life data between each group; however, the weekly collection of EQ-5D-5L data allowed for an accurate estimate of the recovery profile of patients, capturing the speedier improvement of those in the prednisolone group, suggesting that they 'felt better' more quickly than those taking the placebo. In this trial there were low levels of missing data, however when estimating cost at a number of time points over the whole trial period any missing resource use data pose a threat. From the NHS perspective, there were 332 complete cases representing 83% of the total sample who provided data at all time points. The total amount of missing data points was less than this which is borne out by the results of the multiple imputation, confirming that the complete cases estimates are robust. The size of the sample for the trial was based on the primary clinical outcomes, as is common practice. Patient variability affected the uncertainty around the estimates of mean cost, so although there were suggestions of differences between the groups (especially for time off work), the sample was not large enough for this to be confirmed beyond chance. The economic evaluation was restricted by the procedures and time frame of the trial. We did not include a cost for the placebo medication but it is possible that in a 'real life' situation an alternative treatment such as a codeine linctus, an inhaler, or an oral antibiotic might be prescribed. Although it was appropriate to limit the follow up period to 28 days to answer the clinical question we were unable to capture any long term effects of either repeated use of corticosteroids, such as illness medicalisation or osteoporosis,[28] or a reduction in repeated days off work. Finally, no probabilistic sensitivity analysis was performed in this study. Despite other types of sensitivity analyses were performed to account for the variability in the estimates, a global measure of the uncertainty would have been useful in

For peer review only

order to confirm the robustness of the whole model. Besides, a willingness to pay curve would also add value by showing the willingness of these patients to invest in QALY gains.

Comparison with other literature (strengths and weaknesses)

We are aware of no other studies investigating the cost-effectiveness of corticosteroids for ALRTI. However, our estimates of cost and quality of life can be compared separately with other literature. Oppong et al.[29] conducted a thorough analysis of resource use and cost for acute cough/LRTI in 13 European countries. The use of primary healthcare services in that study was slightly higher than our figure of 0.28 per participant: they found a mean of 0.34 visits for the two UK centres included in the study, and an overall range of 0.30 to 1.89. However this difference is small and may be accounted for by different inclusion criteria. On the other hand, the amount of time off work was considerably different. Our participants reported a mean length of 1.16 days off work compared to 3.08 in the European study, though it is difficult to tell whether those authors included a value (representing 'potential' time off) for participants not in paid work. Only one third of our participants reported any time off work but for those who did, the mean length was 3.9 days. In this study we found a small but significant improvement in quality of life for patients taking prednisolone compared with those taking the placebo. In a recent review of short course oral steroids for chronic rhinosinusitis[30] an improvement in quality of life was observed at the end of a two to three week course though the quality of this evidence was judged to be low. There is also some evidence of patients reporting positive mood change when taking steroids.[31]

For peer review only - http://bmjopen.bmj.com/site/about/guidelines.xhtml

Meaning of the study/Policy implications

The results of this study pose an interesting challenge in terms of interpretation. Despite the negative clinical (cough duration and symptom severity) endpoint results, we found prednisolone was cheaper (in terms of NHS costs) than placebo and better (in terms of QALY gain), but each of these findings comes with a caveat. Once the benefit of prescription payments was removed from the analysis the cost gain was much reduced, and could have been due to chance. Placebo patients consistently used more healthcare services than those on prednisolone but as the trial was not powered to detect a difference in cost, we cannot draw any firm conclusions. Although the short term use of prednisolone may make patients feel a little better, the negative effects of longer term or repeated use, such as illness medicalisation[32] and osteoporosis[28] cannot be accounted for in this analysis and are potentially powerful arguments against more widespread use.

The results of this study evidence a small increment of the quality of life - particularly in pain/discomfort and resumption of usual activities -in patients who take prednisolone to treat their ALRTI symptoms. However, taking the clinical and cost-effectiveness results together, and considering the possibility of side-effects from repeated short-term use, the benefits may not outweigh the unknown long term risk.

Unanswered questions and future research

This study has addressed the question of the short term use of corticosteroids for ALRTI but more work is needed to understand the longer term influences. One motivation for carrying out the research was concern about inappropriate prescribing of antibiotics for conditions such as ALRTI, particularly with respect to the effect on antimicrobial resistance. Corticosteroids are successfully used in the short term for many conditions and the long

For peer review only - http://bmjopen.bmj.com/site/about/guidelines.xhtml

term detrimental effects, such as osteoporosis, high blood pressure, and suppression of the immune system are recognised; a recent study [33] showed that adverse events can develop within one month of short-term steroid use in patients with acute respiratory tract infections; however, the specific long term effect of repeated short term use of steroids by ALRTI patients is uncertain. Future work could include long term modelling of the costs and effects of alternative treatments for ALRTI, including corticosteroids, antibiotics, and other medications with little or marginal effect such as linctus. The modelling could take a broad perspective, including the impact of lost productivity, and address the challenge of weighing up the societal cost of antimicrobial resistance against patient level side-effects of medication and the potential for over-medicalisation of ALRTI. In some studies[34,35], the small gains in time to recovery provided by antibiotics (12 to 24 hours) was not sufficient to outweigh their risks. Our study did not measure this variable; however, future research would be necessary to ascertain whether a similar conclusion could be obtained for prednisolone.

Conclusion

The economic evaluation evidences gains in quality of life provided by the use of prednisolone for non-asthmatic adults with ALRTI from the perspective of the NHS. However, the benefit is small and taking the clinical and cost-effectiveness results together, and considering the potential side effects from repeated use of corticosteroids, the short-term benefits may not outweigh the long term harms.

For peer review only - http://bmjopen.bmj.com/site/about/guidelines.xhtml

For peer review only

Funding Statement

This work was supported by the NIHR School for Primary Care Research (grant reference 117a). ADH was funded by NIHR Research Professorship (NIHR-RP-02-12-012). The views expressed are those of the authors and not necessarily those of the NHS, the NIHR or UK Department of Health. A Trial Steering Committee provided independent supervision on behalf of the funder and sponsor (University of Bristol) and an independent Data Monitoring Committee oversaw safety. Ethical approval was granted by the Central Bristol Research Ethics Committee (12/SW/0180). The trial was registered (EudraCT 2012-000851-15 and ISRCTN57309858).

Acknowledgements

We wish to than participants, the recruiting primary care sites, the Clinical Research Networks, and all members of the OSAC team. We would also like to thank the Trial Steering and Data Monitoring Committee members, Nottingham University Hospitals NHS Trust pharmacy and the University Hospitals Bristol NHS Foundation Trust.

Competing Interests Statement

Conflict of Interest Disclosures: All authors have completed and submitted the ICMJE Form for Disclosure of Potential Conflicts of Interest. Dr Thompson reports that he has received funding from Alere Inc to conduct research on C-reactive protein point-of-care tests, has received funding from Roche Molecular Diagnostics for consultancy work.

No other disclosures were reported.

Data Sharing Statement

All the OSAC trial data are available on request and only for academic purpose. Please contact Ms. Grace Young (grace.young@bristol.ac.uk) for data requests.

For peer review only - http://bmjopen.bmj.com/site/about/guidelines.xhtml

Authors' contribution

AH was the Chief Investigator of the OSAC trial; SH and FC designed the economic evaluation; AMF carried out the economic analysis under the supervision of SH. HD was trial manager; MMay designed the statistical analysis which was carried out by GY and SB. AMF, SH, FC HD, GY, SB, MM, MEG, AH, DK, NL, PL, MM, EO, MT, DT, KW and AH contributed to the interpretation of the results. AMF wrote the first draft of the paper. SH, FC, HD, GY, SB, MM, MEG, AH, DK, NL, PL, MM, EO, MT, DT, KW and AH reviewed the manuscript and approved the final version.

Patient and public involvement

Patient participation and involvement (PPI) input was important in the decision to prioritise the research question for funding, and their views informed discussions around the design of the study, the sample size calculation and selection of primary and secondary outcomes. PPI views were sought on recruitment methods, and all patient facing trial materials. The burden of the intervention was also assessed by patients themselves and results were disseminated to both practices and the patients. We wish to thank our PPI advisors for their input into trial design and management.

REFERENCES

1. McCormick A, Fleming D, Charlton J: Morbidity Statistics from General Practice. Fourth National Study 1991-1992. HMSO, London; 1995.

2. Little P, Rumsby K, Kelly J, Watson L, Moore M, Warner G: Information Leaflet and Antibiotic Prescribing Strategies for Acute Lower Respiratory Tract Infection: A Randomised Controlled Trial. JAMA 2005, 293(24):3029-3035.

3. Hollinghurst S, Gorst C, Fahey T, Hay AD. Measuring the financial burden of acute cough in pre-school children: a cost of illness study. BMC Family Practice 2008, 9(10).

4. Schroder K, Fahey T. Systematic Review of Randomised Controlled Trials of over the counter cough medicines for acuet cough in adults. Br Med J 2002, 324(7333):329.

For peer review only - http://bmjopen.bmj.com/site/about/guidelines.xhtml

5. Morice A, McGarvey L, Pavord I. Recommendations for the management of cough in adults. Thorax 2006, 61(supp; 1):i1 - i24.

6. National Institute for Health and Care Excellence (NICE). Respiratory tract infections – antibiotic prescribing. Prescribing of antibiotics for self-limiting respiratory tract infections in adults and children in primary care. In. London; 2008.

7. Thomas M. The management of acute lower respiratory tract infection in adults in primary care. Primary Care Respiratory Journal 2000, 9:4.

8. Huchon G, Gialdroni-Grassi G, Leophonte P, Manresa F, Schaberg T, Woodhead M. Initial antibiotic therapy for lower respiratory tract infection in the community: a European survey. Euro Respir J 1996, 9(8):1590-1595.

9. Adock M, Barnes P. Molecular mechanisms of corticosteroid resistance. Chest 2008, 134(2):394-401.

10. Ponsioen B, Hop W, Vermure N, Dekhuijzen P, Bohnenm AM. Efficacy of fluticasone on cough: a randomised controlled trial. Eur Respir J 2005, 25(1):147-152.

11. Raherison C, Peray P, Poirier R, Romand P, Grignet J, Arsac P. Management of lower respiratory tract infections by French general practitioners: the AIR II study. Analyse Infections Respiratories. Eur Respir J 2002, 19(2):314-319.

12. Ebell MHR, T. Antibiotic use for viral acute respiratory tract infections remains common. The American Journal of Managed Care 2015; 21(10): 9.

13. Downing H, Carroll FE, Brookes S, Hollinghurst S, Timmins D, Orton E, Wang K, Kendrick D, Little P, Moore M: Can oral corticosteroids reduce the severity or duration of an acute

For peer review only - http://bmjopen.bmj.com/site/about/guidelines.xhtml

cough, and the associated National Health Service and societal costs, in adults presenting to primary care? Study protocol for a randomised controlled trial. Trials 2005, 16(1):1-12.

14. Hay AD et al.: Effect of Oral Prednisolone on Symptom Duration and Severity in Nonasthmatic Adults With Acute Lower Respiratory Tract Infection: A Randomized Clinical Trial. JAMA. 2017 Aug 22;318(8):721-730.

15. The EQ-5D-5L http://www.euroqol.org/ [accessed 02/02/2017]

16. Coast J: Is economic evaluation in touch with society's health values? BMJ 2004(329):1233-1236.

17. Curtis L: Unit costs of health and social care 2013, PSSRU, University of Kent http://www.pssru.ac.uk/project-pages/unit-costs/2013 [accessed 02/02/2017]

18. Turner J, O'Cathain A, Knowles E, Nicholl J, Tosh J, Sampson F, Coleman P, Coster J. Evaluation of NHS 111 Pilot sites. Final Report. University of Sheffield; 2012.

19. NHS Reference costs http://www.dh.gov.uk/en/Publicationsandstatistics/Publications/PublicationsPolicyAndGuidance/DH_111591 [accessed 02/02/2017]

20. The AA schedule of motoring costs. http://wwwtheaacom/motoring_advice/running_costs/indexhtml. 2005. [accessed 02/02/2017]

21. Office for National Statistics. Annual survey of hours and earnings 2013, London, 2014

22. British National Formulary 2013. Joint Formulary Committee.

23. NHS Digital. Prescription Cost Analysis, England - 2013.

For peer review only - http://bmjopen.bmj.com/site/about/guidelines.xhtml

24. Manca A, Hawkins N, Sculpher M. Estimating mean QALYs in trial-based cost-effectiveness analysis: the importance of controlling for baseline utility. Health Economics 2005, 14(5):487-496.

25. Efron B, Tibshirani R. An introduction to the bootstrap (Monographs on Statistics and Applied Probability). New York: Chapman & Hall; 1993.

26. StataCorp: Statistical Software: Release 11. College Station, TX: StataCorp LP, 2009.

27. Welch G, Bartlett J, Petersen I. Application of multiple imputation using the two-fold fully conditional specification algorithm in longitudinal clinical data. Stata J 2014, 14(2):418-431.

28. Reid I. Glucocorticoid osteoporosis – mechanisms and management. European Journal of Endocrinology 1997, 137:209-217.

29. Oppong R, Kaambwa B, Nuttall J, Hood K, Smith RD, Coast J: Assessment of the Construct Validity of the EQ-5D in Patients with Acute Cough/Lower Respiratory Tract Infections. Applied Research in Quality of Life 2011, 6(4):411-423.

30. Head K, Chong L, Hopkins C, Philpott C, Burton M, Schider A. Short-course oral steroids alone for chronic rhinosinusitis. Cochrane Databse of Systematic Reviews 2016, 4:CD011991.

31. Sherwood-Brown E, Chandler P. Mood and Cognitive Changes During Systematic Corticosteroid Therapy. Primary Care Companion J Clin Psychiatry 2001, 3(1):17-21.

32. Little P, Williamson I, Warner G, Gould C, Gantley M, Kinmonth A. Open randomised trial of prescribing strategies in managing sore throat. . Br Med J 1997, 314:722-727.

For peer review only

For peer review only - http://bmjopen.bmj.com/site/about/guidelines.xhtml

33. Dvorin EL, Lamb MC, Monlezun DJ, Boese AC, Bazzano LA, Price-Haywood EG. High Frequency of Systemic Corticosteroid Use for Acute Respiratory Tract Illnesses in Ambulatory Settings. JAMA Intern Med. 2018;178(6):852–854.

34. Smucny J, Fahey T, Becker L, et al. Antibiotics for acute bronchitis.Cochrane Database Syst Rev 2000;(4):CD000245.

35. Little P, Rumsby K, Kelly J, et al. Information leaflet and antibiotic prescribing strategies for acute lower respiratory tract infection: a randomized controlled trial. JAMA 2005;293:3029e35.

For peer review only

Table 1. Valuation of resource use

Category of Resource	Unit Cost 2013/14
**Primary Care Services**	
GP (face to face in practice)	46.00[a]
GP (telephone call)	28.00[a]
GP (out of hours)	68.30[a]
Practice nurse (face to face in practice)	13.69[a]
Practice nurse (telephone call)	9.00[a]
Nurse practitioner(face to face in practice)	25.00[a]
Nurse practitioner (out of hours)	33.41[a]
NHS 111 calls	8.29[b]
**Hospital and Walk-in Services**	
Walk-in centre	40.50[c]
Outpatient	150.00/214.00[d]*
A&E visits	111.20[c]
Investigations (X-ray)	28.01[c]
**Medication**	
Prescribed medication	By item[d,e]
Prednisolone (intervention)	1.96[d,e]
Over the counter medication	By item
Prescription charge	7.85
**Other**	
Mileage	0.64[f]
Time off work	118.24[g]

[a] Curtis [17]
[b] Evaluation report [18]
[c] NHS reference costs [19]
[d] BNF [22]
[e] Prescription Pricing Authority [23]
[f] AA schedule [20]
[g] Office of National Statistics [21]

*£214.00 was used for a patient who had a very resource-intensive outpatient stay (upper bound outpatient cost)

For peer review only - http://bmjopen.bmj.com/site/about/guidelines.xhtml

Table 2. Mean (SD) resource use, per patient, by category and group (all available data)

	Prednisolone		Placebo		Unadjusted difference
**Resource use category**	**n**	**Mean (SD)**	**n**	**Mean (SD)**	**Mean (95% CI)**
Primary care consultations	195	0.27 (0.58)	198	0.30 (0.73)	-0.03 (-0.16 to 0.10)
Prescribed medication	190	0.15 (0.51)	200	0.22 (0.64)	-0.06 (-0.18 to 0.05)
NHS 111 calls	177	0.04 (0.22)	168	0.02 (0.13)	0.02 (-0.02 to 0.06)
X-ray procedures	197	0.04 (0.19)	198	0.04 (0.20)	0.00 (-0.04 to 0.03)
All hospital visits	176	0.02 (0.13)	166	0.04 (0.23)	-0.03 (-0.06 to 0.01)
Trial medication	198	1 (0)	200	0 (0)	1 (1 to 1)
Prescription payments	191	0.62 (0.66)	181	0.09 (0.39)	0.52 (0.41 to 0.64)
Over the counter medication	180	0.48 (0.67)	168	0.59 (0.83)	-0.11 (-0.26 to 0.05)
Time off work (days)	171	0.95 (2.61)	165	1.38 (3.05)	-0.43 (-1.04 to 0.18)

For peer review only

For peer review only - http://bmjopen.bmj.com/site/about/guidelines.xhtml

Table 3. Mean (SD) cost (£) by group, per patient, by category and group (all available data)

	Prednisolone		Placebo		Unadjusted difference
**Resource use category**	**n**	**Mean (SD)**	**n**	**Mean (SD)**	**Mean (95%CI)**
Primary care consultations	195	10.38 (23.97)	198	13.11 (33.52)	-2.73 (-8.52 to 3.05)
Prescribed medication	195	0.36 (1.27)	194	0.44 (1.33)	-0.08 (-0.34 to 0.17)
NHS 111 calls	168	0.33 (1.85)	177	0.33 (1.85)	0.18 (-0.14 to 0.50)
X-ray procedures	198	1.00 (5.20)	197	1.00 (5.20)	-0.14 (-1.20 to 0.93)
All hospital visits	176	1.09 (9.39)	166	3.84 (23.10)	-2.75 (-6.46 to 0.97)
Trial medication	198	1.96 (0)	200	0 (0)	1.96 (1.96 to 1.96)
Prescription payments[a]	191	4.85 (5.20)	181	0.74 (3.06)	4.11 (3.24 to 4.99)
Over the counter medication	175	2.23 (3.93)	165	3.08 (5.07)	-0.85 (-1.82 to 0.11)
Travel costs	171	1.76 (2.63)	166	2.45 (4.39)	-0.69 (-1.47 to 0.08)
Time off work (days)	161	58.02 (161.16)	160	83.88 (183.93)	-25.86 (-63.83 to 12.11)

[a] Prescription payments are a transfer cost between the NHS and patients. They are a negative cost (receipt) to the NHS and a positive cost to patients

For peer review only

Table 4. Cost-consequence analysis. Differences in mean (95%CI) cost and Quality Adjusted Life Years (complete cases, by perspective)

	Mean (95% CI)[a] Difference
All NHS services (n=332)	-£7.00 (-£17.08 to -£0.50)
All patient out-of-pocket expenditure (n=329)	£2.90 (£1.14 to £4.48)
Value of time off work (n=321)	-£30.45 (-£ 67.15 to £9.79)
QALYs (n=346)	0.03 (0.01, 0.05)
Duration of cough (n=334)	Hazard Ratio: 1.11 (0.89 to 1.39)
Symptom severity score (n=368)	-0.20 (-0.40 to 0.00)

[a] *biased corrected and adjusted by centre and baseline covariates*

For peer review only

Table 5. Sensitivity Analyses: incremental costs and QALYs under different scenarios.

SCENARIO	Differences in means* (95% CI)
1. Imputed data (ICE)	
NHS services	-£9.78 (-£19.05 to -£3.87)
Patient out-of-pocket expenditure	£3.01 (£1.06 to £4.28)
Value of time off work	-£22.99 (-£55.81 to £12.97)
QALYs	0.03 (0.01 to 0.04)
2. Imputed data (TWOFOLD)	
NHS services	-£7.27 (-£17.12 to -£0.91)
Patient out-of-pocket expenditure	£2.97 (£1.16 to £4.54)
Value of time off work	-£28.36 (-£67.75 to £9.38)
QALYs	0.03 (0.01 to 0.04)
3. Exclusion of potentially unrelated cost categories (n=332)	
NHS services	-£6.20 (-£13.77 to £0.14)
4. QALYs using VAS (n=326)	
QALYs	0.01 (-0.01 to 0.03)
5. Removal of prescription payments (n=334)	
NHS services	-£3.04 (-£11.31 to £5.24)
Patient out-of-pocket expenditure	-£1.49 (-£2.83 to -£0.16)

**adjusted by centre and baseline covariates*

For peer review only - http://bmjopen.bmj.com/site/about/guidelines.xhtml

Appendix

Table A. Participants' baseline characteristics

	Prednisolone (N=198)	Placebo (N=200)
Centre, n (%)		
Bristol	118 (60%)	113 (57%)
Oxford	39 (20%)	45 (23%)
Southampton	24 (12%)	21 (11%)
Nottingham	17 (9%)	21 (11%)
Demographics and past medical history		
Gender, n (%) male	82 (41%)	66 (33%)
Age, mean (SD)	50.0 (16.1)	44.8 (15.5)
Weight kg, median (IQR)[a]	77.0 (64.5,91.0)	76.0 (66.5,90.5)
Height cm, median (IQR)[b]	168.0 (161.0,175.0)	168.0 (163.0,176.0)
Ethnicity, n (%) white[c]	188 (95%)	193 (97%)
Occupation, n (%)		
Employed	137 (69%)	143 (72%)
Unemployed	17 (9%)	21 (11%)
Retired	41 (21%)	30 (15%)
Full-time education	3 (2%)	6 (3%)
Deprivation (IMD)[d], median (IQR)[e]	11.0 (5.0,23.0)	12.0 (5.0,23.0)
Smoking status, n (%)[f]		
Current	31 (16%)	38 (19%)
Past	63 (32%)	55 (28%)
Never	104 (53%)	106 (53%)
Lives with smoker, n (%)[g]	25 (14%)	32 (16%)
Received asthma medication >5 years previously[h]	10 (5%)	8 (4%)
Personal history of hay fever[i]	41 (22%)	46 (24%)
Personal history of eczema[j]	30 (16%)	26 (14%)
Family history asthma/hay fever/eczema, n (%)[k]	73 (40%)	76 (40%)
Influenza vaccine in last 12 months, n (%)	63 (32%)	44 (22%)
Recruited in winter (1st Oct-31st March)	112 (57%)	114 (57%)
Clinical characteristics and management		
Prior duration of cough, median (IQR) days	13.0 (7.0,20.0)	10.0 (6.0,17.5)
Sputum (symptom <24hr), n (%)[l]	149 (76%)	156 (78%)
Shortness of breath (symptom <24hr) n (%)	146 (74%)	133 (67%)

(Table continues on next page)

For peer review only - http://bmjopen.bmj.com/site/about/guidelines.xhtml

Table A. Participants' baseline characteristics *(cont.)*

	Prednisolone (N=198)	Placebo (N=200)
Wheeze (symptom <24hr), n (%)[l]	88 (45%)	98 (49%)
Chest pain (symptom <24hr) n (%)	88 (44%)	97 (49%)
Patient reported illness severity (0-10), median (IQR)[m]	6.0 (5.0,7.0)	5.0 (4.0,7.0)
Pulse rate (bpm), mean (SD)	77.8 (12.3)	77.7 (11.8)
Temperature (^{o}C), mean (SD)	36.6 (0.5)	36.6 (0.4)
Oxygen saturation (%), mean (SD)[n]	97.5 (1.3)	97.8 (1.1)
Baseline abnormal peak flow[o]	87 (44%)	79 (40%)
Abnormal respiratory rate, n (%)	2 (1%)	1 (1%)
Chest recession/prolonged expiration	0 (0%)	1 (1%)
Wheeze/rhonchi (auscultation), n (%)	11 (6%)	11 (6%)
Crackles/crepitations (auscultation), n (%)[p]	4 (2%)	6 (3%)
Bronchial breathing	0 (0%)	2 (1%)
Taken prescribed β agonist in past 24 hours, n (%)	9 (5%)	3 (2%)
OTC[q] drugs taken for current cough, n (%)	128 (65%)	139 (70%)
Given delayed antibiotic script, n (%)	22 (11%)	25 (13%)

[a] Weight missing for 2 prednisolone participants
[b] Height missing for 1 prednisolone participant
[c] Ethnicity missing for 1 placebo participant
[d] English Index of Multiple Deprivation scores (2015) [Geoconvert: UK Data Service Census Support]
[e] IMD missing for 2 prednisolone and 7 placebo participants
[f] Smoking status missing for 1 placebo participant
[g] Living with smoker missing for 15 prednisolone and 5 placebo participants
[h] Personal history of asthma missing for 10 prednisolone patients and 7 placebo patients
[I] Personal history of hayfever missing for 10 prednisolone patients and 11 placebo patients
[j] Personal history of eczema missing for 14 prednisolone patients and 10 placebo patients
[k] Family history of hay fever/eczema/asthma missing for 16 prednisolone and 11 placebo participants
[l] Sputum and wheeze presence in 24 hours, missing for 1 prednisolone participant
[m] Patient reported illness severity measured on zero to 10 scale, missing for 1 prednisolone participant
[n] Oxygen saturation missing for 1 prednisolone participant
[o] Baseline abnormal peak flow (defined as <80% of expected peak flow) was missing for 1 prednisolone patient
[p] Includes unilateral and bilateral
[q] Over-the-counter

For peer review only - http://bmjopen.bmj.com/site/about/guidelines.xhtml

Table B. Percentage of patients reporting no problems in response to the five domains of the EQ-5D

		**Baseline – all participants** (n=199 placebo; 198 prednisolone)	**Baseline – participants with week 4 data** (n=171 placebo; 177 prednisolone)	**Week 4** (n=171 placebo; 177 prednisolone)
Mobility	Placebo	93.0	94.1	94.7
	Prednisolone	91.9	91.5	94.4
Self-care	Placebo	97.5	97.7	97.1
	Prednisolone	99.0	98.9	99.4
Usual activities	Placebo	89.9	90.0	83.0
	Prednisolone	82.3	82.5	93.2
Pain/discomfort	Placebo	61.3	62.9	81.3
	Prednisolone	55.6	54.8	87.6
Anxiety/depression	Placebo	83.4	84.1	86.5
	Prednisolone	82.3	82.5	89.3

For peer review only

CHEERS Checklist
Items to include when reporting economic evaluations of health interventions

The **ISPOR CHEERS Task Force Report**, *Consolidated Health Economic Evaluation Reporting Standards (CHEERS)—Explanation and Elaboration: A Report of the ISPOR Health Economic Evaluations Publication Guidelines Good Reporting Practices Task Force,* provides examples and further discussion of the 24-item CHEERS Checklist and the CHEERS Statement. It may be accessed via the *Value in Health* or via the ISPOR Health Economic Evaluation Publication Guidelines – CHEERS: Good Reporting Practices webpage: http://www.ispor.org/TaskForces/EconomicPubGuidelines.asp

Section/item	Item No	Recommendation	Reported on page No/ line No
**Title and abstract**			
Title	1	Identify the study as an economic evaluation or use more specific terms such as "cost-effectiveness analysis", and describe the interventions compared.	1
Abstract	2	Provide a structured summary of objectives, perspective, setting, methods (including study design and inputs), results (including base case and uncertainty analyses), and conclusions.	2
**Introduction**			
Background and objectives	3	Provide an explicit statement of the broader context for the study. Present the study question and its relevance for health policy or practice decisions.	4-5
**Methods**			
Target population and subgroups	4	Describe characteristics of the base case population and subgroups analysed, including why they were chosen.	5
Setting and location	5	State relevant aspects of the system(s) in which the decision(s) need(s) to be made.	5
Study perspective	6	Describe the perspective of the study and relate this to the costs being evaluated.	6
Comparators	7	Describe the interventions or strategies being compared and state why they were chosen.	5
Time horizon	8	State the time horizon(s) over which costs and consequences are being evaluated and say why appropriate.	6
Discount rate	9	Report the choice of discount rate(s) used for costs and outcomes and say why appropriate.	9
Choice of health outcomes	10	Describe what outcomes were used as the measure(s) of benefit in the evaluation and their relevance for the type of analysis performed.	5
Measurement of effectiveness	11a	*Single study-based estimates:* Describe fully the design features of the single effectiveness study and why the single study was a sufficient source of clinical effectiveness data.	5

	11b	*Synthesis-based estimates:* Describe fully the methods used for identification of included studies and synthesis of clinical effectiveness data.	N/A
Measurement and valuation of preference based outcomes	12	If applicable, describe the population and methods used to elicit preferences for outcomes.	8
Estimating resources and costs	13a	*Single study-based economic evaluation:* Describe approaches used to estimate resource use associated with the alternative interventions. Describe primary or secondary research methods for valuing each resource item in terms of its unit cost. Describe any adjustments made to approximate to opportunity costs.	7/8
	13b	*Model-based economic evaluation:* Describe approaches and data sources used to estimate resource use associated with model health states. Describe primary or secondary research methods for valuing each resource item in terms of its unit cost. Describe any adjustments made to approximate to opportunity costs.	N/A
Currency, price date, and conversion	14	Report the dates of the estimated resource quantities and unit costs. Describe methods for adjusting estimated unit costs to the year of reported costs if necessary. Describe methods for converting costs into a common currency base and the exchange rate.	7
Choice of model	15	Describe and give reasons for the specific type of decision-analytical model used. Providing a figure to show model structure is strongly recommended.	6
Assumptions	16	Describe all structural or other assumptions underpinning the decision-analytical model.	6/9
Analytical methods	17	Describe all analytical methods supporting the evaluation. This could include methods for dealing with skewed, missing, or censored data; extrapolation methods; methods for pooling data; approaches to validate or make adjustments (such as half cycle corrections) to a model; and methods for handling population heterogeneity and uncertainty.	6/9
**Results**			
Study parameters	18	Report the values, ranges, references, and, if used, probability distributions for all parameters. Report reasons or sources for distributions used to represent uncertainty where appropriate. Providing a table to show the input values is strongly recommended.	9-12
Incremental costs and outcomes	19	For each intervention, report mean values for the main categories of estimated costs and outcomes of interest, as well as mean differences between the comparator groups. If applicable, report incremental cost-effectiveness ratios.	25-28
Characterising uncertainty	20a	*Single study-based economic evaluation:* Describe the effects of sampling uncertainty for the estimated incremental cost and incremental effectiveness parameters, together with the impact	12-13

		of methodological assumptions (such as discount rate, study perspective).	N/A
	20b	*Model-based economic evaluation:* Describe the effects on the results of uncertainty for all input parameters, and uncertainty related to the structure of the model and assumptions.	N/A
Characterising heterogeneity	21	If applicable, report differences in costs, outcomes, or cost-effectiveness that can be explained by variations between subgroups of patients with different baseline characteristics or other observed variability in effects that are not reducible by more information.	13-17
**Discussion**			
Study findings, limitations, generalisability, and current knowledge	22	Summarise key study findings and describe how they support the conclusions reached. Discuss limitations and the generalisability of the findings and how the findings fit with current knowledge.	18
**Other**			
Source of funding	23	Describe how the study was funded and the role of the funder in the identification, design, conduct, and reporting of the analysis. Describe other non-monetary sources of support.	18
Conflicts of interest	24	Describe any potential for conflict of interest of study contributors in accordance with journal policy. In the absence of a journal policy, we recommend authors comply with International Committee of Medical Journal Editors recommendations.	

For consistency, the CHEERS Statement checklist format is based on the format of the CONSORT statement checklist

The **ISPOR CHEERS Task Force Report** provides examples and further discussion of the 24-item CHEERS Checklist and the CHEERS Statement. It may be accessed via the *Value in Health* link or via the ISPOR Health Economic Evaluation Publication Guidelines – CHEERS: Good Reporting Practices webpage: http://www.ispor.org/TaskForces/EconomicPubGuidelines.asp

The citation for the CHEERS Task Force Report is:
Husereau D, Drummond M, Petrou S, et al. Consolidated health economic evaluation reporting standards (CHEERS)—Explanation and elaboration: A report of the ISPOR health economic evaluations publication guidelines good reporting practices task force. Value Health 2013;16:231-50.

BMJ Open

Economic evaluation of the OSAC randomised controlled trial: oral corticosteroids for non-asthmatic adults with acute lower respiratory tract infection in primary care

Journal:	*BMJ Open*
Manuscript ID	bmjopen-2019-033567.R1
Article Type:	Original research
Date Submitted by the Author:	18-Sep-2019
Complete List of Authors:	Moure-Fernandez, Aide; Hospital Clínic de Barcelona,<br>Hollinghurst, Sandra; University of Bristol, Community Based Medicine<br>Carroll, Fran; University of Bristol, School of Social and Community Medicine<br>Downing, Harriet; University of Bristol, Community Based Medicine<br>Young, Grace; University of Bristol, School of Social and Community Medicine; Bristol Randomised Trials Collaboration, University of Bristol<br>Brookes, Sara ; University of Bristol,<br>May, Margaret; University of Bristol, School of Social and Community Medicine<br>El-Gohary, Magdy; University of Southampton, 2) Department of Primary Care and Population Sciences<br>Harnden, Anthony; Oxford University, Dept of Primary Health Care<br>Kendrick, Denise; University of Nottingham<br>Lafond, Natasher; University of Nottingham School of Medicine<br>Little, Paul; University of Southampton, Primary Care and Population Science<br>Moore, Michael; University of Southampton Medical School, Primary Care Medical Group<br>Orton, Elizabeth; University of Nottingham<br>Thompson, Matthew; University of Washington, Department of Family Medicine<br>Timmins, David; Oxford Brookes University Faculty of Health and Life Sciences, NIHR School for Primary Care Research; Nuffield department of primary care health sciences<br>Wang, Kay; Nuffield Department of Primary Care Health Sciences<br>Hay, Alastair; University of Bristol, Centre for Academic Primary Care
**Primary Subject Heading**:	Health economics
Secondary Subject Heading:	Respiratory medicine
Keywords:	HEALTH ECONOMICS, RESPIRATORY MEDICINE (see Thoracic Medicine), cost-effectiveness, ALRTI, PRIMARY CARE

For peer review only - http://bmjopen.bmj.com/site/about/guidelines.xhtml

Economic evaluation of the OSAC randomised controlled trial: oral corticosteroids for non-asthmatic adults with acute lower respiratory tract infection in primary care.

Aída Moure Fernández; Sandra Hollinghurst; Fran Carroll; Harriet Downing; Grace Young; Sara Brookes; Margaret May; Magdy El-Gohary; Anthony Harnden; Denise Kendrick; Natasher Lafond; Paul Little; Michael Moore; Elizabeth Orton; Matthew Thompson; David Timmins; Kay Wang; Alastair D Hay.

Author Affiliations: Centre for Academic Primary Care, National Institute for Health Research (NIHR) School for Primary Care Research, Population Health Sciences, Bristol Medical School, University of Bristol, Bristol, England (Hay, Hollinghurst, Downing); Primary Care and Population Science, NIHR School for Primary Care Research, Faculty of Medicine, University of Southampton, Aldermoor Health Center, Southampton, England (Little, El-Gohary, Moore); Nuffield Department of Primary Care Health Sciences, NIHR School for Primary Care Research, University of Oxford, Oxford, England (Harnden, Wang, Timmins); Department of Family Medicine, University of Washington, Seattle (Thompson); Division of Primary Care, NIHR School for Primary Care Research, School of Medicine, University of Nottingham, Nottingham, England (Kendrick, Orton, Lafond); Population Health Sciences, Bristol Medical School, University of Bristol, Bristol, England (Brookes, May); Bristol Randomised Trials Collaboration, Population Health Sciences, Bristol Medical School, University of Bristol, Bristol, England (Young); Oblikue Consulting, SL (Moure Fernández); Translational Health Sciences, Bristol Medical School, University of Bristol, Bristol, England (Carroll).

Corresponding Author: Aída Moure Fernández. Hospital Clínic de Barcelona. Villarroel, 170. 08036 Barcelona, Spain. E-mail:amoure@clinic.cat/ aidamoure@gmail.com

For peer review only
For peer review only - http://bmjopen.bmj.com/site/about/guidelines.xhtml

Abstract

Objective

To estimate the costs and outcomes associated with treating non-asthmatic (nor suffering from other lung-disease) adults presenting to primary care with acute lower respiratory tract infection (ALRTI) with oral corticosteroids compared with placebo.

Design

Cost-consequence analysis alongside a randomised controlled trial. Perspectives included the healthcare provider, patients, and productivity losses associated with time off-work.

Setting

Fifty-four NHS general practices in England.

Participants

398 adults attending NHS primary practices with ALRTI but no asthma or other chronic lung disease, followed up for 28 days.

Interventions

2x20mg oral prednisolone daily for five days versus matching placebo tablets.

Outcome measures

Quality-adjusted life years using the EQ-5D-5L measured weekly; duration and severity of symptom. Direct and indirect resources related to the disease and its treatment were also collected. Outcomes were measured for the 28-day follow up.

Results

198 (50%) patients received the intervention (prednisolone) and 200 (50%) received placebo. NHS costs were dominated by primary care contacts, higher with placebo than with prednisolone (£13.11 vs £10.38) but without evidence of a difference (95% CI: -£3.05 to £8.52). The trial medication cost of £1.96 per patient would have been recouped in

For peer review only

prescription charges of £4.30 per patient overall (55% participants would have paid £7.85), giving an overall mean ‘profit’ to the NHS of £7.00 (95% CI: £0.50 to £17.08) per patient. There was a QALY gain of 0.03 (95% CI: 0.01 to 0.05) equating to half a day of perfect health favouring the prednisolone patients; there was no difference in duration of cough or severity of symptoms.

Conclusions

The use of prednisolone for non-asthmatic adults with ALRTI, provided small gains in quality of life and cost savings driven by prescription charges. Considering the results of the economic evaluation and possible side effects of corticosteroids, the short-term benefits may not outweigh the long term harms.

For peer review only

Strengths and limitations of this study

- The economic evaluation was part of a rigorously conducted multicentre randomised controlled trial, involving a representative population of patients not thought to need immediate antibiotic treatment.
- The economic evaluation included the perspectives of patients and time off work as well as the NHS.
- Low levels of missing cost and outcome data, with EQ-5D observations from multiple time points, achieving an accurate profile of patient health-related quality of life over the period of the illness.
- The analysis was thorough and included multiple imputation of missing data and extensive sensitivity analysis.

For peer review only - http://bmjopen.bmj.com/site/about/guidelines.xhtml

Economic evaluation of the OSAC randomised controlled trial: oral corticosteroids for non-asthmatic adults with acute lower respiratory tract infection in primary care.

INTRODUCTION

Acute lower respiratory tract infection (ALRTI), with symptoms such as wheeze, phlegm, and chest pain, is one of the most common reasons for patients to consult in primary care.[1] In the UK ALRTI costs the National Health Service at least £190 million annually,[2] and further costs are borne by patients in self-managing their condition[3, 4] and by society in general because of work absenteeism.[5]

Despite lack of evidence of efficacy and the recommendation from the National Institute for Health and Care Excellence of a non-antibiotic pathway,[6] antibiotics are still frequently prescribed to treat ALRTI.[7, 8] This unnecessary prescribing fuels antimicrobial resistance, raises patient expectations for similar treatment in the future (so-called 'illness medicalisation'), and is a wasteful use of healthcare resources.

Oral and inhaled corticosteroids are widely used to treat symptoms of asthma[9] and there is some evidence of the effectiveness of high doses of inhaled corticosteroids in reducing cough frequency among non-smokers with ALRTI who do not have asthma or other chronic lung disease.[10] Given the similarity in symptoms of ALRTI and those of asthma, we tested the hypothesis that corticosteroids might be an effective treatment for ALRTI in adults without asthma or other chronic lung disease. Indeed, there is increasing evidence from Europe[11] and the United States[12] of steroid prescribing for patients with ALRTI, with a secondary analysis of one US study[12] showing 15% of non-asthmatic adults with ALRTI being prescribed oral steroids.

For peer review only

The OSAC study[13,14] examined the effectiveness and costs of oral corticosteroids (more specifically oral prednisolone) in treating ALRTI in a non-asthmatic adult population compared with placebo. Here, we report on the results of the economic evaluation.

METHODS

The OSAC study was a two-arm randomised controlled trial that aimed to test the provision of oral corticosteroids to adults with ALRTI but no asthma or other chronic lung disease such as chronic obstructive pulmonary disease. Details of the trial procedures and clinical results are reported elsewhere.[13,14] Briefly, patients with an ALRTI-related cough were recruited from 54 primary care practices across four areas of England and randomly allocated to receive either oral prednisolone (intervention) or placebo (control) for their acute cough symptoms. The intervention consisted of 40mg (two 20mg tablets) of prednisolone daily for five days. The control group received matching placebo tablets. The primary clinical outcomes of the study were duration of “moderately bad or worse” cough, and symptom severity for days 2 to 4 post randomisation. The primary outcome for the economic evaluation was quality adjusted life years (QALYs) which were estimated from responses to the EQ-5D-5L.[15]

Design

The economic evaluation was conducted from the perspectives of the NHS, patients, and a broader perspective taking into account differences in work-related productivity costs; we included all resource use during the 28-day follow up period. We used a cost-consequences

For peer review only - http://bmjopen.bmj.com/site/about/guidelines.xhtml

design,[16] comparing cost from the three perspectives with the clinical outcomes and QALYs, in order to see all possible costs and health consequences.

Identification of resources

NHS resources were identified as being: the trial medication, primary care consultations, other relevant prescribed medication, hospital care, use of NHS 111, and ALRTI-related investigations such as chest X-ray. Patient resource use was identified as being: travel, prescription costs, and over-the-counter medication and remedies. Productivity losses were identified as being days off work due to the cough.

Measurement of resources: data collection

A primary care notes review took place at the end of the study period and data were extracted on: all primary care consultations, categorised as doctor or nurse, face to face, telephone or home visit, and in-hours or out-of-hours; prescribed medication; and investigations. Participants in the study were issued with a daily diary to complete, which was used to collect data on health service use not always reliably available from primary care notes such as use of NHS 111 and hospital services. Participant out-of-pocket expenses on travel and over-the-counter medicines were also recorded here, along with time off work. Participants were telephoned weekly to reinforce their record keeping.

Valuation of resources

Resources were valued as shown in Table 1. All resources were costed in pounds sterling at 2013-2014 prices, using an appropriate inflation index[17] when necessary. Costs for primary care services were obtained from Curtis;[17] calls to the NHS 111 service were

For peer review only

costed using a published national evaluation[18] and NHS reference costs[19] were used to value secondary care services. Travelling by car was valued using the AA running schedule to cost the mileage,[20] and standard ticket prices were employed to cost the use of public transport. Productivity costs were derived using the age/sex average rate from the Annual Survey of Hours and Earnings.[21]

For prescribed medication, costs were extracted mainly from the British National Formulary (BNF).[22] A cost was obtained for each prescription based on the name of the drug, the method of administration (for example, tablet, capsule, liquid) and the number of units included in the prescription. When the cost of a drug was not available in the BNF, the Prescription Costs Analysis of England database[23] was used to cost that medication. Uncertainties around relevance (to ALRTI) and missing information were resolved by seeking clinical advice.

Careful consideration was given to the most accurate way of costing the intervention medication (prednisolone) with the aim of reflecting the true cost to the NHS if it were to be adopted as a strategy for treating ALRTI. First, thought was given to the number of pills that would be needed to cover the intervention total dose as the 20mg tablets provided in the study are not routinely available. We assumed that 5mg tablets would be a reasonable substitute, resulting in 8 tablets required per day, and 40 tablets for the duration of the intervention (5 days). From the basic price of £1.03 given in the BNF, adjustments were made as follows. We subtracted the usual discount (7.61% of the basic price) eligible to the dispensing pharmacy from the manufacturer; we added a standard 90p dispensing fee and a payment of 1.24p for consumables; and as the amount required was 40 and packs contain

For peer review only - http://bmjopen.bmj.com/site/about/guidelines.xhtml

28 tablets, an extra 10p for splitting packs was added. This resulted in a cost for the intervention prednisolone of £1.96. To reflect a 'roll-out' situation, we allowed for potential receipts from prescription payments made by patients by including an amount for those participants who reported that they usually pay a prescription charge.

No cost was included for the placebo medication (i.e. tablets) as this was a research cost.

Measurement and valuation of outcomes

The EQ-5D-5L was completed weekly by participants from baseline during the 28-day follow up, giving us five observations to use to form QALYs. Utilities were obtained from existing preferences elicited from the general public, using the crosswalk algorithm.[15] QALYs were calculated from these utilities using the area under the curve and adjusting for baseline differences.[24]

Results from the clinical trial [14] show that the median duration of moderately bad or worse cough was 5 days in both groups and the hazard ratio of 1.11 (95% CI: 0.89 to 1.39) also indicates no difference. There was a reduction of 0.02 in mean symptom score for days 2 to 4 in prednisolone patients though this was less than the pre-determined clinically meaningful difference.

Data analysis

Frequencies of resource use were calculated to provide a descriptive analysis of the types of resources used by primary care patients with a cough. We estimated mean resource use and cost per patient for all categories, by trial arm, to compare the prednisolone group with those using the placebo. Ordinary least squares regressions were employed to calculate the differences in costs adjusted for centre, age, gender and outcome-related baseline

For peer review only

variables, in order to account for potential imbalances between the groups. Standard deviations (for means) and bias-corrected bootstrapped confidence intervals[25] (2,000 replicates) were constructed to account for the uncertainty in the point estimates.

No discount was applied to the data as the time horizon of the study was 28 days. All analyses were carried out using Stata 13 and above.[26]

For peer review only

Sensitivity Analyses

Five different sensitivity analyses were conducted to test the robustness of assumptions made in the base case analysis. The effect of missing data was appraised in two scenarios using multiple imputation techniques: in addition to the imputation with chained equations (ICE) (scenario 1), which is usually employed in economic evaluations, an analysis was also carried out using the 'twofold' command in Stata; this command imputes missing values at a given time point, conditional on information at the same time point and immediately adjacent time points [27] (scenario 2), to be consistent with the methodology used in the clinical effectiveness analysis.[14] Scenario 3 excluded outpatient and A&E attendances where there was any ambiguity about their relevance to LRTI; scenario 4 used the visual analogue scale to calculate QALYs; and scenario 5 removed the prescription payments from the analysis on the basis that these are to some extent artefactual and relate specifically to England at the time of the study.

Patient and public involvement

Patient participation and involvement (PPI) input was important in the decision to prioritise the research question for funding, and their views informed discussions around the design of the study,

For peer review only - http://bmjopen.bmj.com/site/about/guidelines.xhtml

the sample size calculation and selection of primary and secondary outcomes. PPI views were sought on recruitment methods, and all patient facing trial materials. The burden of the intervention was also assessed by patients themselves and results were disseminated to both practices and the patients. We wish to thank our PPI advisors for their input into trial design and management.

RESULTS

In total, 398 patients were included in the intention-to-treat analysis. One hundred and ninety eight (49.7%) were in the trial medication (intervention) group and 200 (50.3%) received the matched placebo (control). Baseline characteristics of the participants are shown in Table A of the appendix. Participants were predominantly white, employed, and middle-aged and similar rates received asthma medication in both groups (5% vs. 4%) during the previous years. Approximately 50% in both arms stated they had never smoked. Data on NHS costs were complete for 332 (83%) participants and out-of-pocket costs for patients were reported by 329 (83%). Time off work was recorded by 321 (81%) of the participants filling the questionnaire and 346 (87%) completed the EQ-5D-5L at all five time points.

Resource use

Use of all health services was relatively modest during the 28 day follow up period. Overall just 20% participants accessed primary care, a proportion that was similar for both groups though patients in the placebo group had slightly more consultations per patient than those in the prednisolone group, which is reflected in the mean number of encounters (0.30 vs 0.27) as shown in Table 2. Prescribed medication was also slightly higher in the placebo group in terms of number of patients (15% vs 12%) being prescribed anything and the mean

For peer review only

number of prescriptions per participant (0.22 vs 0.15). Nine participants (2%) reported using hospital services: three (1.5%) in the prednisolone group and six (3%) in the placebo group.

More placebo group participants reported buying over-the-counter medications than those in the prednisolone group (45% vs 39%) and the mean number of items per participant was higher (0.59 vs 0.48). Popular items included cough linctus and cold & flu remedies. Similarly, more placebo group participants reported time off work than prednisolone participants (32% vs 28%) and the mean length of time off was longer (1.38 days vs 0.95 days).

Although there was a consistently higher use of resources in the placebo group compared with the prednisolone group there is imprecision around the point estimates and, with the exception of the trial medication and associated prescription payments, there is no evidence of a difference in use between the groups as indicated by the confidence intervals reported in Table 2.

Cost analysis

Table 3 summarises the cost comparison between the two groups. The majority of NHS costs were attributable to primary care consultations with hospital visits, including for X-rays, contributing a modest amount. Over half (55%) of prednisolone participants reported that they normally pay a prescription charge; the value of prescription payments made by these patients more than covered the cost of the trial medication meaning that the NHS on balance would have made a profit. Out-of-pocket patient costs were dominated by prescription payments and the value of time off work was higher than any other category of

For peer review only - http://bmjopen.bmj.com/site/about/guidelines.xhtml
For peer review only

cost. Comparing the prednisolone and placebo groups, costs were higher in the placebo group for all categories (except those related to the trial medication) though again there was no evidence of a difference between the groups as indicated by the confidence intervals.

Cost-consequence analysis

Table 4 shows the comparison of incremental costs and outcomes, including the results from the clinical trial regarding duration of cough and symptom severity score. Here we present the difference in cost by perspective, adjusted by centre, age, gender, and baseline covariates. The 95% confidence intervals are bootstrapped and bias corrected. The negative incremental cost of -£7.00 (95% CI: -£17.08 to -£0.50) per patient to the NHS indicates the intervention is cheaper than placebo; it reflects the lower use of primary care by patients in the prednisolone group and the offset of intervention costs by prescription payments. From the patient perspective, despite higher travel and over the counter costs in the placebo group, total costs were approximately £3 more in the prednisolone group due to the cost of prescriptions. The value of time off work was approximately £30 higher per patient for the placebo group.

The gain in quality of life provided by the prednisolone was 0.03 QALYs (95% CI: 0.01 to 0.05) per patient, which translates into slightly more than half a day of extra ‘best imaginable’ health during the 28 days. The percentage of patients reporting no problems for each domain of the EQ-5D are shown in table B of the Appendix. On average, the prednisolone patients improved by more than the placebo patients in all domains but the

For peer review only

‘pain and discomfort’ and ‘usual activities’ domains were where the greatest difference was seen.

Sensitivity analysis

Results of the sensitivity analyses are presented in Table 5. Imputing missing data, and excluding unrelated costs make no difference to the conclusions of the base case analysis. Using the visual analogue scale (VAS) to value the QALYs reduced the QALY gain of prednisolone patients over placebo to a minimal amount (0.01: 95% CI: -0.01 to 0.03). When prescription payments are removed the incremental cost to the NHS is still negative (-£3.04; 95% CI: -£11.31 to £5.24) though the confidence interval suggests no evidence of a true difference. The reverse is true for patient costs as placebo patients spent considerable more on travel and over-the-counter medications and remedies than those in the prednisolone group.

DISCUSSION

Summary of main findings

Prednisolone was found to be clinically ineffective for treating ALRTI in non-asthmatic adults in primary care in terms of duration of cough and symptom severity [14]; however there was evidence that patients using prednisolone experienced a greater improvement in health-related quality of life than those using the placebo. This was largely due to a greater improvement in pain/discomfort and a speedier return to carrying out usual activities. Prednisolone is a relatively inexpensive medication and, in this population of generally healthy adults ineligible for prescription charge exemption, prescription payments more than offset the cost to the NHS. Other NHS resource use was consistently higher in the

For peer review only - http://bmjopen.bmj.com/site/about/guidelines.xhtml

placebo group across all categories though the differences were small. The value of time off work was considerable and this was higher in the placebo patients, though the limited sample size combined with patient-level variation prevented a robust conclusion.

Strengths and weaknesses

The sample included in OSAC was typical of patients consulting in primary care with ALRTI, not thought to require immediate antibiotic treatment. The two groups were well matched at baseline and there was minimal drop-out. The economic evaluation was carried out at individual patient level and covered the whole 28 day period. Data on actual utility values for each group was not reported, which would have helped to understand the differences regarding quality of life data between each group; however, the weekly collection of EQ-5D-5L data allowed for an accurate estimate of the recovery profile of patients, capturing the speedier improvement of those in the prednisolone group, suggesting that they 'felt better' more quickly than those taking the placebo. In this trial there were low levels of missing data, however when estimating cost at a number of time points over the whole trial period any missing resource use data pose a threat. From the NHS perspective, there were 332 complete cases representing 83% of the total sample who provided data at all time points. The total amount of missing data points was less than this which is borne out by the results of the multiple imputation, confirming that the complete cases estimates are robust. The size of the sample for the trial was based on the primary clinical outcomes, as is common practice. Patient variability affected the uncertainty around the estimates of mean cost, so although there were suggestions of differences between the groups (especially for time off work), the sample was not large enough for this to be confirmed beyond chance. The economic evaluation was restricted by the procedures and time frame of the trial. We did

For peer review only

not include a cost for the placebo medication but it is possible that in a ‘real life’ situation an alternative treatment such as a codeine linctus, an inhaler, or an oral antibiotic might be prescribed. Although it was appropriate to limit the follow up period to 28 days to answer the clinical question we were unable to capture any long term effects of either repeated use of corticosteroids, such as illness medicalisation or osteoporosis,[28] or a reduction in repeated days off work.

Comparison with other literature (strengths and weaknesses)

We are aware of no other studies investigating the clinical and economic implications of corticosteroids for ALRTI. However, our estimates of cost and quality of life can be compared separately with other literature. Oppong et al.[29] conducted a thorough analysis of resource use and cost for acute cough/LRTI in 13 European countries. The use of primary healthcare services in that study was slightly higher than our figure of 0.28 per participant: they found a mean of 0.34 visits for the two UK centres included in the study, and an overall range of 0.30 to 1.89. However this difference is small and may be accounted for by different inclusion criteria. On the other hand, the amount of time off work was considerably different. Our participants reported a mean length of 1.16 days off work compared to 3.08 in the European study, though it is difficult to tell whether those authors included a value (representing ‘potential’ time off) for participants not in paid work. Only one third of our participants reported any time off work but for those who did, the mean length was 3.9 days. In this study we found a small but significant improvement in quality of life for patients taking prednisolone compared with those taking the placebo. In a recent review of short course oral steroids for chronic rhinosinusitis[30] an improvement in quality of life was observed at the end of a two to three week course though the quality of this

For peer review only - http://bmjopen.bmj.com/site/about/guidelines.xhtml

evidence was judged to be low. There is also some evidence of patients reporting positive mood change when taking steroids.[31]

Meaning of the study/Policy implications

The results of this study pose an interesting challenge in terms of interpretation. Despite the negative clinical (cough duration and symptom severity) endpoint results, we found prednisolone was cheaper (in terms of NHS costs) than placebo and better (in terms of QALY gain), but each of these findings comes with a caveat. Once the benefit of prescription payments was removed from the analysis the cost gain was much reduced, and could have been due to chance. Placebo patients consistently used more healthcare services than those on prednisolone but as the trial was not powered to detect a difference in cost, we cannot draw any firm conclusions. Although the short term use of prednisolone may make patients feel a little better, the negative effects of longer term or repeated use, such as illness medicalisation[32] and osteoporosis[28] cannot be accounted for in this analysis and are potentially powerful arguments against more widespread use.

The results of this study evidence a small increment of the quality of life - particularly in pain/discomfort and resumption of usual activities -in patients who take prednisolone to treat their ALRTI symptoms. However, taking the clinical and economic implications derived from this study, and considering the possibility of side-effects from repeated short-term use, the benefits may not outweigh the unknown long term risk.

Unanswered questions and future research

This study has addressed the question of the short term use of corticosteroids for ALRTI but more work is needed to understand the longer term influences. One motivation for carrying

For peer review only

out the research was concern about inappropriate prescribing of antibiotics for conditions such as ALRTI, particularly with respect to the effect on antimicrobial resistance. Corticosteroids are successfully used in the short term for many conditions and the long term detrimental effects, such as osteoporosis, high blood pressure, and suppression of the immune system are recognised; a recent study [33] showed that adverse events can develop within one month of short-term steroid use in patients with acute respiratory tract infections; however, the specific long term effect of repeated short term use of steroids by ALRTI patients is uncertain. Future work could include long term modelling of the costs and effects of alternative treatments for ALRTI, including corticosteroids, antibiotics, and other medications with little or marginal effect such as linctus. The modelling could take a broad perspective, including the impact of lost productivity, and address the challenge of weighing up the societal cost of antimicrobial resistance against patient level side-effects of medication and the potential for over-medicalisation of ALRTI. In some studies[34,35], the small gains in time to recovery provided by antibiotics (12 to 24 hours) was not sufficient to outweigh their risks. Our study did not measure this variable; however, future research would be necessary to ascertain whether a similar conclusion could be obtained for prednisolone.

Conclusion

The economic evaluation evidences gains in quality of life provided by the use of prednisolone for non-asthmatic adults with ALRTI from the perspective of the NHS. However, the benefit is small and taking the clinical and economic implications derived from this study, and considering the potential side effects from repeated use of corticosteroids, the short-term benefits may not outweigh the long term harms.

For peer review only - http://bmjopen.bmj.com/site/about/guidelines.xhtml

For peer review only

Funding Statement

This work was supported by the NIHR School for Primary Care Research (grant reference 117a). ADH was funded by NIHR Research Professorship (NIHR-RP-02-12-012). The views expressed are those of the authors and not necessarily those of the NHS, the NIHR or UK Department of Health. A Trial Steering Committee provided independent supervision on behalf of the funder and sponsor (University of Bristol) and an independent Data Monitoring Committee oversaw safety. Ethical approval was granted by the Central Bristol Research Ethics Committee (12/SW/0180). The trial was registered (EudraCT 2012-000851-15 and ISRCTN57309858).

Acknowledgements

We wish to than participants, the recruiting primary care sites, the Clinical Research Networks, and all members of the OSAC team. We would also like to thank the Trial Steering and Data Monitoring Committee members, Nottingham University Hospitals NHS Trust pharmacy and the University Hospitals Bristol NHS Foundation Trust.

Competing Interests Statement

Conflict of Interest Disclosures: All authors have completed and submitted the ICMJE Form for Disclosure of Potential Conflicts of Interest. Dr Thompson reports that he has received funding from Alere Inc to conduct research on C-reactive protein point-of-care tests, has received funding from Roche Molecular Diagnostics for consultancy work.

No other disclosures were reported.

Data Sharing Statement

All the OSAC trial data are available on request and only for academic purpose. Please contact Ms. Grace Young (grace.young@bristol.ac.uk) for data requests.

For peer review only

Authors' contribution

AH was the Chief Investigator of the OSAC trial; SH and FC designed the economic evaluation; AMF carried out the economic analysis under the supervision of SH. HD was trial manager; MMay designed the statistical analysis which was carried out by GY and SB. AMF, SH, FC HD, GY, SB, MM, MEG, AH, DK, NL, PL, MM, EO, MT, DT, KW and AH contributed to the interpretation of the results. AMF wrote the first draft of the paper. SH, FC, HD, GY, SB, MM, MEG, AH, DK, NL, PL, MM, EO, MT, DT, KW and AH reviewed the manuscript and approved the final version.

REFERENCES

1. McCormick A, Fleming D, Charlton J: Morbidity Statistics from General Practice. Fourth National Study 1991-1992. HMSO, London; 1995.
2. Little P, Rumsby K, Kelly J, Watson L, Moore M, Warner G: Information Leaflet and Antibiotic Prescribing Strategies for Acute Lower Respiratory Tract Infection: A Randomised Controlled Trial. JAMA 2005, 293(24):3029-3035.
3. Hollinghurst S, Gorst C, Fahey T, Hay AD. Measuring the financial burden of acute cough in pre-school children: a cost of illness study. BMC Family Practice 2008, 9(10).
4. Schroder K, Fahey T. Systematic Review of Randomised Controlled Trials of over the counter cough medicines for acuet cough in adults. Br Med J 2002, 324(7333):329.
5. Morice A, McGarvey L, Pavord I. Recommendations for the management of cough in adults. Thorax 2006, 61(supp; 1):i1 - i24.
6. National Institute for Health and Care Excellence (NICE). Respiratory tract infections – antibiotic prescribing. Prescribing of antibiotics for self-limiting respiratory tract infections in adults and children in primary care. In. London; 2008.
7. Thomas M. The management of acute lower respiratory tract infection in adults in primary care. Primary Care Respiratory Journal 2000, 9:4.

For peer review only - http://bmjopen.bmj.com/site/about/guidelines.xhtml

For peer review only

8. Huchon G, Gialdroni-Grassi G, Leophonte P, Manresa F, Schaberg T, Woodhead M. Initial antibiotic therapy for lower respiratory tract infection in the community: a European survey. Euro Respir J 1996, 9(8):1590-1595.

9. Adock M, Barnes P. Molecular mechanisms of corticosteroid resistance. Chest 2008, 134(2):394-401.

10. Ponsioen B, Hop W, Vermure N, Dekhuijzen P, Bohnenm AM. Efficacy of fluticasone on cough: a randomised controlled trial. Eur Respir J 2005, 25(1):147-152.

11. Raherison C, Peray P, Poirier R, Romand P, Grignet J, Arsac P. Management of lower respiratory tract infections by French general practitioners: the AIR II study. Analyese Infections Respiratories. Eur Respir J 2002, 19(2):314-319.

12. Ebell MHR, T. Antibiotic use for viral acute respiratory tract infections remains common. The American Journal of Managed Care 2015; 21(10): 9.

13. Downing H, Carroll FE, Brookes S, Hollinghurst S, Timmins D, Orton E, Wang K, Kendrick D, Little P, Moore M: Can oral corticosteroids reduce the severity or duration of an acute cough, and the associated National Health Service and societal costs, in adults presenting to primary care? Study protocol for a randomised controlled trial. Trials 2005, 16(1):1-12.

14. Hay AD et al.: Effect of Oral Prednisolone on Symptom Duration and Severity in Nonasthmatic Adults With Acute Lower Respiratory Tract Infection: A Randomized Clinical Trial. JAMA. 2017 Aug 22;318(8):721-730.

15. The EQ-5D-5L http://www.euroqol.org/ [accessed 02/02/2017]

16. Coast J: Is economic evaluation in touch with society's health values? BMJ 2004(329):1233-1236.

17. Curtis L: Unit costs of health and social care 2013, PSSRU, University of Kent http://www.pssru.ac.uk/project-pages/unit-costs/2013 [accessed 02/02/2017]

For peer review only

18. Turner J, O'Cathain A, Knowles E, Nicholl J, Tosh J, Sampson F, Coleman P, Coster J. Evaluation of NHS 111 Pilot sites. Final Report. University of Sheffield; 2012.

19. NHS Reference costs http://www.dh.gov.uk/en/Publicationsandstatistics/Publications/PublicationsPolicyAndGuidance/DH_111591 [accessed 02/02/2017]

20. The AA schedule of motoring costs. http://wwwtheaacom/motoring_advice/running_costs/indexhtml. 2005. [accessed 02/02/2017]

21. Office for National Statistics. Annual survey of hours and earnings 2013, London, 2014

22. British National Formulary 2013. Joint Formulary Committee.

23. NHS Digital. Prescription Cost Analysis, England - 2013.

24. Manca A, Hawkins N, Sculpher M. Estimating mean QALYs in trial-based cost-effectiveness analysis: the importance of controlling for baseline utility. Health Economics 2005, 14(5):487-496.

25. Efron B, Tibshirani R. An introduction to the bootstrap (Monographs on Statistics and Applied Probability). New York: Chapman & Hall; 1993.

26. StataCorp: Statistical Software: Release 11. College Station, TX: StataCorp LP, 2009.

27. Welch G, Bartlett J, Petersen I. Application of multiple imputation using the two-fold fully conditional specification algorithm in longitudinal clinical data. Stata J 2014, 14(2):418-431.

28. Reid I. Glucocorticoid osteoporosis – mechanisms and management. European Journal of Endocrinology 1997, 137:209-217.

29. Oppong R, Kaambwa B, Nuttall J, Hood K, Smith RD, Coast J: Assessment of the Construct Validity of the EQ-5D in Patients with Acute Cough/Lower Respiratory Tract Infections. Applied Research in Quality of Life 2011, 6(4):411-423.

For peer review only - http://bmjopen.bmj.com/site/about/guidelines.xhtml

30. Head K, Chong L, Hopkins C, Philpott C, Burton M, Schider A. Short-course oral steroids alone for chronic rhinosinusitis. Cochrane Databse of Systematic Reviews 2016, 4:CD011991.

31. Sherwood-Brown E, Chandler P. Mood and Cognitive Changes During Systematic Corticosteroid Therapy. Primary Care Companion J Clin Psychiatry 2001, 3(1):17-21.

32. Little P, Williamson I, Warner G, Gould C, Gantley M, Kinmonth A. Open randomised trial of prescribing strategies in managing sore throat. . Br Med J 1997, 314:722-727.

33. Dvorin EL, Lamb MC, Monlezun DJ, Boese AC, Bazzano LA, Price-Haywood EG. High Frequency of Systemic Corticosteroid Use for Acute Respiratory Tract Illnesses in Ambulatory Settings. JAMA Intern Med. 2018;178(6):852–854.

34. Smucny J, Fahey T, Becker L, et al. Antibiotics for acute bronchitis.Cochrane Database Syst Rev 2000;(4):CD000245.

35. Little P, Rumsby K, Kelly J, et al. Information leaflet and antibiotic prescribing strategies for acute lower respiratory tract infection: a randomized controlled trial. JAMA 2005;293:3029e35.

For peer review only

Table 1. Valuation of resource use

Category of Resource	Unit Cost 2013/14
**Primary Care Services**	
GP (face to face in practice)	46.00[a]
GP (telephone call)	28.00 [a]
GP (out of hours)	68.30 [a]
Practice nurse (face to face in practice)	13.69 [a]
Practice nurse (telephone call)	9.00 [a]
Nurse practitioner(face to face in practice)	25.00 [a]
Nurse practitioner (out of hours)	33.41 [a]
NHS 111 calls	8.29[b]
**Hospital and Walk-in Services**	
Walk-in centre	40.50[c]
Outpatient	150.00/214.00[d]*
A&E visits	111.20 [c]
Investigations (X-ray)	28.01 [c]
**Medication**	
Prescribed medication	By item[d,e]
Prednisolone (intervention)	1.96[d,e]
Over the counter medication	By item
Prescription charge	7.85
**Other**	
Mileage	0.64[f]
Time off work	118.24[g]

[a] Curtis [17]
[b] Evaluation report [18]
[c] NHS reference costs [19]
[d] BNF [22]
[e] Prescription Pricing Authority [23]
[f] AA schedule [20]
[g] Office of National Statistics [21]

*£214.00 was used for a patient who had a very resource-intensive outpatient stay (upper bound outpatient cost)

For peer review only - http://bmjopen.bmj.com/site/about/guidelines.xhtml

Table 2. Mean (SD) resource use, per patient, by category and group (all available data)

	Prednisolone		Placebo		Unadjusted difference
**Resource use category**	**n**	**Mean (SD)**	**n**	**Mean (SD)**	**Mean (95% CI)**
Primary care consultations	195	0.27 (0.58)	198	0.30 (0.73)	-0.03 (-0.16 to 0.10)
Prescribed medication	190	0.15 (0.51)	200	0.22 (0.64)	-0.06 (-0.18 to 0.05)
NHS 111 calls	177	0.04 (0.22)	168	0.02 (0.13)	0.02 (-0.02 to 0.06)
X-ray procedures	197	0.04 (0.19)	198	0.04 (0.20)	0.00 (-0.04 to 0.03)
All hospital visits	176	0.02 (0.13)	166	0.04 (0.23)	-0.03 (-0.06 to 0.01)
Trial medication	198	1 (0)	200	0 (0)	1 (1 to 1)
Prescription payments	191	0.62 (0.66)	181	0.09 (0.39)	0.52 (0.41 to 0.64)
Over the counter medication	180	0.48 (0.67)	168	0.59 (0.83)	-0.11 (-0.26 to 0.05)
Time off work (days)	171	0.95 (2.61)	165	1.38 (3.05)	-0.43 (-1.04 to 0.18)

Table 3. Mean (SD) cost (£) by group, per patient, by category and group (all available data)

	Prednisolone		Placebo		Unadjusted difference
**Resource use category**	**n**	**Mean (SD)**	**n**	**Mean (SD)**	**Mean (95%CI)**
Primary care consultations	195	10.38 (23.97)	198	13.11 (33.52)	-2.73 (-8.52 to 3.05)
Prescribed medication	195	0.36 (1.27)	194	0.44 (1.33)	-0.08 (-0.34 to 0.17)
NHS 111 calls	168	0.33 (1.85)	177	0.33 (1.85)	0.18 (-0.14 to 0.50)
X-ray procedures	198	1.00 (5.20)	197	1.00 (5.20)	-0.14 (-1.20 to 0.93)
All hospital visits	176	1.09 (9.39)	166	3.84 (23.10)	-2.75 (-6.46 to 0.97)
Trial medication	198	1.96 (0)	200	0 (0)	1.96 (1.96 to 1.96)
Prescription payments[a]	191	4.85 (5.20)	181	0.74 (3.06)	4.11 (3.24 to 4.99)
Over the counter medication	175	2.23 (3.93)	165	3.08 (5.07)	-0.85 (-1.82 to 0.11)
Travel costs	171	1.76 (2.63)	166	2.45 (4.39)	-0.69 (-1.47 to 0.08)
Time off work (days)	161	58.02 (161.16)	160	83.88 (183.93)	-25.86 (-63.83 to 12.11)

[a] Prescription payments are a transfer cost between the NHS and patients. They are a negative cost (receipt) to the NHS and a positive cost to patients

For peer review only - http://bmjopen.bmj.com/site/about/guidelines.xhtml

For peer review only

Table 4. Cost-consequence analysis. Differences in mean (95%CI) cost and Quality Adjusted Life Years (complete cases, by perspective)

	Mean (95% CI)[a] Difference
All NHS services (n=332)	-£7.00 (-£17.08 to -£0.50)
All patient out-of-pocket expenditure (n=329)	£2.90 (£1.14 to £4.48)
Value of time off work (n=321)	-£30.45 (-£ 67.15 to £9.79)
QALYs (n=346)	0.03 (0.01, 0.05)
Duration of cough (n=334)	Hazard Ratio: 1.11 (0.89 to 1.39)
Symptom severity score (n=368)	-0.20 (-0.40 to 0.00)

[a] *biased corrected and adjusted by centre and baseline covariates*

For peer review only

Table 5. Sensitivity Analyses: incremental costs and QALYs under different scenarios.

SCENARIO	Differences in means* (95% CI)
**1.** Imputed data (ICE)	
NHS services	-£9.78 (-£19.05 to -£3.87)
Patient out-of-pocket expenditure	£3.01 (£1.06 to £4.28)
Value of time off work	-£22.99 (-£55.81 to £12.97)
QALYs	0.03 (0.01 to 0.04)
2. Imputed data (TWOFOLD)	
NHS services	-£7.27 (-£17.12 to -£0.91)
Patient out-of-pocket expenditure	£2.97 (£1.16 to £4.54)
Value of time off work	-£28.36 (-£67.75 to £9.38)
QALYs	0.03 (0.01 to 0.04)
3. Exclusion of potentially unrelated cost categories (n=332)	
NHS services	-£6.20 (-£13.77 to £0.14)
4. QALYs using VAS (n=326)	
QALYs	0.01 (-0.01 to 0.03)
**5.** Removal of prescription payments (n=334)	
NHS services	-£3.04 (-£11.31 to £5.24)
Patient out-of-pocket expenditure	-£1.49 (-£2.83 to -£0.16)

**adjusted by centre and baseline covariates*

For peer review only

Appendix

Table A. Participants' baseline characteristics

	Prednisolone (N=198)	Placebo (N=200)
Centre, n (%)		
Bristol	118 (60%)	113 (57%)
Oxford	39 (20%)	45 (23%)
Southampton	24 (12%)	21 (11%)
Nottingham	17 (9%)	21 (11%)
Demographics and past medical history		
Gender, n (%) male	82 (41%)	66 (33%)
Age, mean (SD)	50.0 (16.1)	44.8 (15.5)
Weight kg, median (IQR)[a]	77.0 (64.5,91.0)	76.0 (66.5,90.5)
Height cm, median (IQR)[b]	168.0 (161.0,175.0)	168.0 (163.0,176.0)
Ethnicity, n (%) white[c]	188 (95%)	193 (97%)
Occupation, n (%)		
Employed	137 (69%)	143 (72%)
Unemployed	17 (9%)	21 (11%)
Retired	41 (21%)	30 (15%)
Full-time education	3 (2%)	6 (3%)
Deprivation (IMD)[d], median (IQR)[e]	11.0 (5.0,23.0)	12.0 (5.0,23.0)
Smoking status, n (%)[f]		
Current	31 (16%)	38 (19%)
Past	63 (32%)	55 (28%)
Never	104 (53%)	106 (53%)
Lives with smoker, n (%)[g]	25 (14%)	32 (16%)
Received asthma medication >5 years previously[h]	10 (5%)	8 (4%)
Personal history of hay fever[i]	41 (22%)	46 (24%)
Personal history of eczema[j]	30 (16%)	26 (14%)
Family history asthma/hay fever/eczema, n (%)[k]	73 (40%)	76 (40%)
Influenza vaccine in last 12 months, n (%)	63 (32%)	44 (22%)
Recruited in winter (1st Oct-31st March)	112 (57%)	114 (57%)
Clinical characteristics and management		
Prior duration of cough, median (IQR) days	13.0 (7.0,20.0)	10.0 (6.0,17.5)
Sputum (symptom <24hr), n (%)[l]	149 (76%)	156 (78%)
Shortness of breath (symptom <24hr) n (%)	146 (74%)	133 (67%)

(Table continues on next page)

For peer review only - http://bmjopen.bmj.com/site/about/guidelines.xhtml

Table A. Participants' baseline characteristics *(cont.)*

	Prednisolone (N=198)	Placebo (N=200)
Wheeze (symptom <24hr), n (%)[l]	88 (45%)	98 (49%)
Chest pain (symptom <24hr) n (%)	88 (44%)	97 (49%)
Patient reported illness severity (0-10), median (IQR)[m]	6.0 (5.0,7.0)	5.0 (4.0,7.0)
Pulse rate (bpm), mean (SD)	77.8 (12.3)	77.7 (11.8)
Temperature (^{o}C), mean (SD)	36.6 (0.5)	36.6 (0.4)
Oxygen saturation (%), mean (SD)[n]	97.5 (1.3)	97.8 (1.1)
Baseline abnormal peak flow[o]	87 (44%)	79 (40%)
Abnormal respiratory rate, n (%)	2 (1%)	1 (1%)
Chest recession/prolonged expiration	0 (0%)	1 (1%)
Wheeze/rhonchi (auscultation), n (%)	11 (6%)	11 (6%)
Crackles/crepitations (auscultation), n (%)[p]	4 (2%)	6 (3%)
Bronchial breathing	0 (0%)	2 (1%)
Taken prescribed β agonist in past 24 hours, n (%)	9 (5%)	3 (2%)
OTC[q] drugs taken for current cough, n (%)	128 (65%)	139 (70%)
Given delayed antibiotic script, n (%)	22 (11%)	25 (13%)

[a] Weight missing for 2 prednisolone participants
[b] Height missing for 1 prednisolone participant
[c] Ethnicity missing for 1 placebo participant
[d] English Index of Multiple Deprivation scores (2015) [Geoconvert: UK Data Service Census Support]
[e] IMD missing for 2 prednisolone and 7 placebo participants
[f] Smoking status missing for 1 placebo participant
[g] Living with smoker missing for 15 prednisolone and 5 placebo participants
[h] Personal history of asthma missing for 10 prednisolone patients and 7 placebo patients
[i] Personal history of hayfever missing for 10 prednisolone patients and 11 placebo patients
[j] Personal history of eczema missing for 14 prednisolone patients and 10 placebo patients
[k] Family history of hay fever/eczema/asthma missing for 16 prednisolone and 11 placebo participants
[l] Sputum and wheeze presence in 24 hours, missing for 1 prednisolone participant
[m] Patient reported illness severity measured on zero to 10 scale, missing for 1 prednisolone participant
[n] Oxygen saturation missing for 1 prednisolone participant
[o] Baseline abnormal peak flow (defined as <80% of expected peak flow) was missing for 1 prednisolone patient
[p] Includes unilateral and bilateral
[q] Over-the-counter

For peer review only - http://bmjopen.bmj.com/site/about/guidelines.xhtml

Table B. Percentage of patients reporting no problems in response to the five domains of the EQ-5D

		**Baseline – all participants** (n=199 placebo; 198 prednisolone)	**Baseline – participants with week 4 data** (n=171 placebo; 177 prednisolone)	**Week 4** (n=171 placebo; 177 prednisolone)
Mobility	Placebo	93.0	94.1	94.7
	Prednisolone	91.9	91.5	94.4
Self-care	Placebo	97.5	97.7	97.1
	Prednisolone	99.0	98.9	99.4
Usual activities	Placebo	89.9	90.0	83.0
	Prednisolone	82.3	82.5	93.2
Pain/discomfort	Placebo	61.3	62.9	81.3
	Prednisolone	55.6	54.8	87.6
Anxiety/depression	Placebo	83.4	84.1	86.5
	Prednisolone	82.3	82.5	89.3

For peer review only

CHEERS Checklist
Items to include when reporting economic evaluations of health interventions

The **ISPOR CHEERS Task Force Report**, *Consolidated Health Economic Evaluation Reporting Standards (CHEERS)—Explanation and Elaboration: A Report of the ISPOR Health Economic Evaluations Publication Guidelines Good Reporting Practices Task Force,* provides examples and further discussion of the 24-item CHEERS Checklist and the CHEERS Statement. It may be accessed via the *Value in Health* or via the ISPOR Health Economic Evaluation Publication Guidelines – CHEERS: Good Reporting Practices webpage: http://www.ispor.org/TaskForces/EconomicPubGuidelines.asp

Section/item	Item No	Recommendation	Reported on page No/ line No
**Title and abstract**			
Title	1	Identify the study as an economic evaluation or use more specific terms such as "cost-effectiveness analysis", and describe the interventions compared.	1
Abstract	2	Provide a structured summary of objectives, perspective, setting, methods (including study design and inputs), results (including base case and uncertainty analyses), and conclusions.	2
**Introduction**			
Background and objectives	3	Provide an explicit statement of the broader context for the study.<br>Present the study question and its relevance for health policy or practice decisions.	4-5
**Methods**			
Target population and subgroups	4	Describe characteristics of the base case population and subgroups analysed, including why they were chosen.	5
Setting and location	5	State relevant aspects of the system(s) in which the decision(s) need(s) to be made.	5
Study perspective	6	Describe the perspective of the study and relate this to the costs being evaluated.	6
Comparators	7	Describe the interventions or strategies being compared and state why they were chosen.	5
Time horizon	8	State the time horizon(s) over which costs and consequences are being evaluated and say why appropriate.	6
Discount rate	9	Report the choice of discount rate(s) used for costs and outcomes and say why appropriate.	9
Choice of health outcomes	10	Describe what outcomes were used as the measure(s) of benefit in the evaluation and their relevance for the type of analysis performed.	5
Measurement of effectiveness	11a	*Single study-based estimates:* Describe fully the design features of the single effectiveness study and why the single study was a sufficient source of clinical effectiveness data.	5

	11b	*Synthesis-based estimates:* Describe fully the methods used for identification of included studies and synthesis of clinical effectiveness data.	N/A
Measurement and valuation of preference based outcomes	12	If applicable, describe the population and methods used to elicit preferences for outcomes.	8
Estimating resources and costs	13a	*Single study-based economic evaluation:* Describe approaches used to estimate resource use associated with the alternative interventions. Describe primary or secondary research methods for valuing each resource item in terms of its unit cost. Describe any adjustments made to approximate to opportunity costs.	7/8
	13b	*Model-based economic evaluation:* Describe approaches and data sources used to estimate resource use associated with model health states. Describe primary or secondary research methods for valuing each resource item in terms of its unit cost. Describe any adjustments made to approximate to opportunity costs.	N/A
Currency, price date, and conversion	14	Report the dates of the estimated resource quantities and unit costs. Describe methods for adjusting estimated unit costs to the year of reported costs if necessary. Describe methods for converting costs into a common currency base and the exchange rate.	7
Choice of model	15	Describe and give reasons for the specific type of decision-analytical model used. Providing a figure to show model structure is strongly recommended.	6
Assumptions	16	Describe all structural or other assumptions underpinning the decision-analytical model.	6/9
Analytical methods	17	Describe all analytical methods supporting the evaluation. This could include methods for dealing with skewed, missing, or censored data; extrapolation methods; methods for pooling data; approaches to validate or make adjustments (such as half cycle corrections) to a model; and methods for handling population heterogeneity and uncertainty.	6/9
**Results**			
Study parameters	18	Report the values, ranges, references, and, if used, probability distributions for all parameters. Report reasons or sources for distributions used to represent uncertainty where appropriate. Providing a table to show the input values is strongly recommended.	9-12
Incremental costs and outcomes	19	For each intervention, report mean values for the main categories of estimated costs and outcomes of interest, as well as mean differences between the comparator groups. If applicable, report incremental cost-effectiveness ratios.	25-28
Characterising uncertainty	20a	*Single study-based economic evaluation:* Describe the effects of sampling uncertainty for the estimated incremental cost and incremental effectiveness parameters, together with the impact	12-13

		of methodological assumptions (such as discount rate, study perspective).	N/A
	20b	*Model-based economic evaluation:* Describe the effects on the results of uncertainty for all input parameters, and uncertainty related to the structure of the model and assumptions.	N/A
Characterising heterogeneity	21	If applicable, report differences in costs, outcomes, or cost-effectiveness that can be explained by variations between subgroups of patients with different baseline characteristics or other observed variability in effects that are not reducible by more information.	13-17
**Discussion**			
Study findings, limitations, generalisability, and current knowledge	22	Summarise key study findings and describe how they support the conclusions reached. Discuss limitations and the generalisability of the findings and how the findings fit with current knowledge.	18
**Other**			
Source of funding	23	Describe how the study was funded and the role of the funder in the identification, design, conduct, and reporting of the analysis. Describe other non-monetary sources of support.	18
Conflicts of interest	24	Describe any potential for conflict of interest of study contributors in accordance with journal policy. In the absence of a journal policy, we recommend authors comply with International Committee of Medical Journal Editors recommendations.	

For consistency, the CHEERS Statement checklist format is based on the format of the CONSORT statement checklist

The **ISPOR CHEERS Task Force Report** provides examples and further discussion of the 24-item CHEERS Checklist and the CHEERS Statement. It may be accessed via the *Value in Health* link or via the ISPOR Health Economic Evaluation Publication Guidelines – CHEERS: Good Reporting Practices webpage: http://www.ispor.org/TaskForces/EconomicPubGuidelines.asp

The citation for the CHEERS Task Force Report is:
Husereau D, Drummond M, Petrou S, et al. Consolidated health economic evaluation reporting standards (CHEERS)—Explanation and elaboration: A report of the ISPOR health economic evaluations publication guidelines good reporting practices task force. Value Health 2013;16:231-50.
